# Modeling of Shear Flows over Superhydrophobic Surfaces: From Newtonian to Non-Newtonian Fluids

**DOI:** 10.1021/acsengineeringau.3c00048

**Published:** 2024-01-04

**Authors:** Hossein Rahmani, Faïçal Larachi, Seyed Mohammad Taghavi

**Affiliations:** Department of Chemical Engineering, Université Laval, Québec, QC, Canada G1 V 0A6

**Keywords:** Superhydrophobic, Shear flow, Newtonian, Non-Newtonian, Mathematical, Numerical, Shear-thinning, Viscoplastic

## Abstract

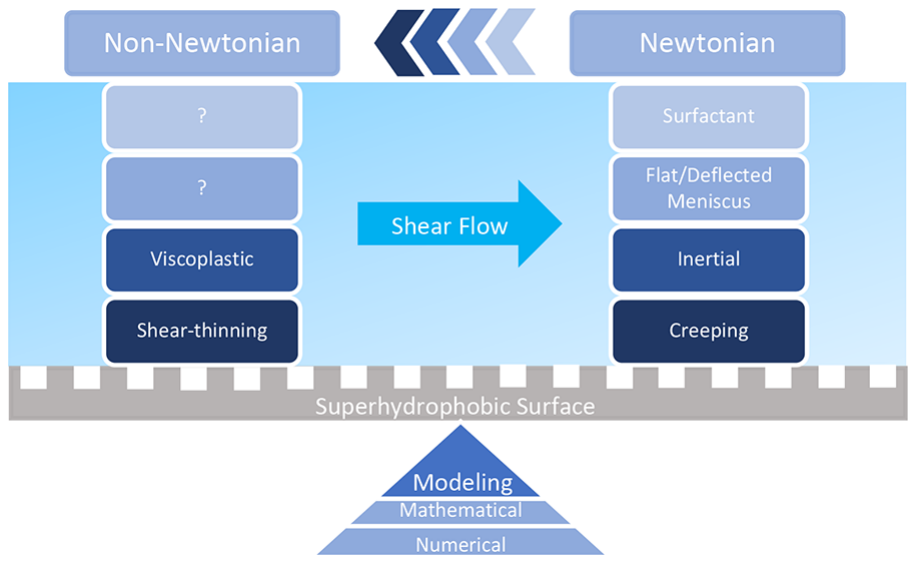

The design and use of superhydrophobic surfaces have gained special attentions due to their superior performances and advantages in many flow systems, e.g., in achieving specific goals including drag reduction and flow/droplet handling and manipulation. In this work, we conduct a brief review of shear flows over superhydrophobic surfaces, covering the classic and recent studies/trends for both Newtonian and non-Newtonian fluids. The aim is to mainly review the relevant mathematical and numerical modeling approaches developed during the past 20 years. Considering the wide ranges of applications of superhydrophobic surfaces in Newtonian fluid flows, we attempt to show how the developed studies for the Newtonian shear flows over superhydrophobic surfaces have been evolved, through highlighting the major breakthroughs. Despite the fact that, in many practical applications, flows over superhydrophobic surfaces may show complex non-Newtonian rheology, interactions between the non-Newtonian rheology and superhydrophobicity have not yet been well understood. Therefore, in this Review, we also highlight emerging recent studies addressing the shear flows of shear-thinning and yield stress fluids in superhydrophobic channels. We focus on reviewing the models developed to handle the intricate interaction between the formed liquid/air interface on superhydrophobic surfaces and the overlying flow. Such an intricate interaction will be more complex when the overlying flow shows nonlinear non-Newtonian rheology. We conclude that, although our understanding on the Newtonian shear flows over superhydrophobic surfaces has been well expanded via analyzing various aspects of such flows, the non-Newtonian counterpart is in its early stages. This could be associated with either the early applications mainly concerning Newtonian fluids or new complexities added to an already complex problem by the nonlinear non-Newtonian rheology. Finally, we discuss the possible directions for development of models that can address complex non-Newtonian shear flows over superhydrophobic surfaces.

## Introduction

**Figure 1:**
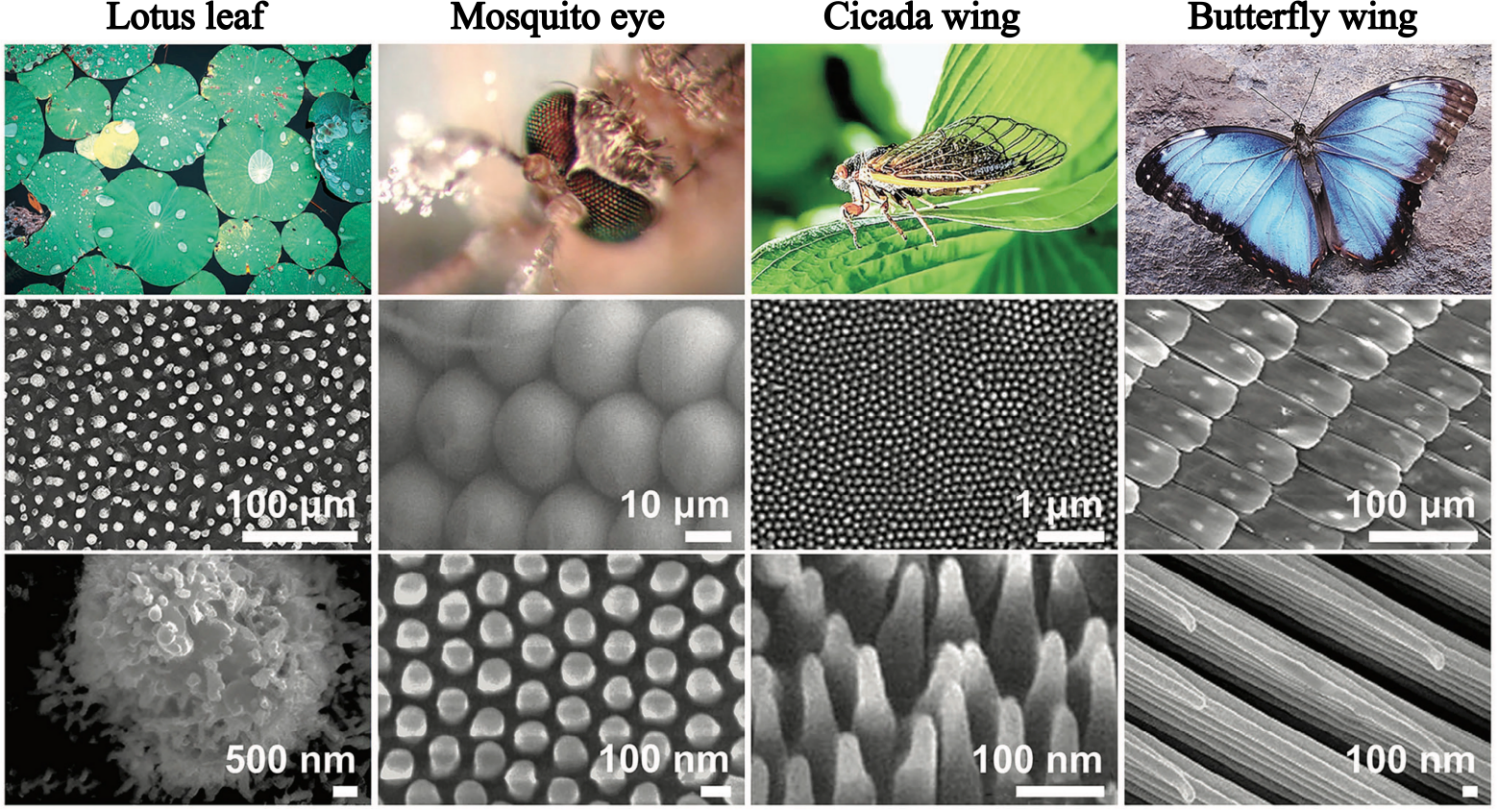
Examples of superhydrophobic surfaces in nature. For each surface, the micro and nano scale protrusions are illustrated. Adapted from Tang et al.^13^ with permission. Copyright 2023 John Wiley and Sons.

When fluid flows over a surface, the characteristics of the surface itself can play a significant role in the flow dynamics.^1−6^ Even small-scale features on the surface can result in major alterations on the flow physics. For instance, inspired by properties of biosurfaces, e.g. lotus leaves, researchers have developed superhydrophobic surfaces^7−9^ (see Figure 1). This involves adding micronano scale protrusions to a given surface, which reduces the surface wettability and consequently enhances its slipperiness.^10,11^ These micronano structures, often in the form of grooves, posts and holes, are deliberately engineered on both hydrophilic and hydrophobic surfaces. The aim is to trap pockets of air within the formed cavities.^7−9^ The trapped air reduces the direct contact between the flowing liquid and the solid surface, creating a partial- or no-shear condition on its interface with the liquid. Such an alteration significantly enhances the slippery nature of the surface.^9,12^

The ideal state at which a liquid sits on a superhydrophobic surface while being separated by trapped air pockets is referred to the *Cassie* state. However, there are instances when the trapped air escapes (or solubilizes in the liquid), and the liquid infiltrates the cavities, leading to the *Wenzel* state.^9,12^ The occurrence of this transition largely depends on the flow properties as well as the characteristics of the surface, such as system pressure, surface tension, and fluid rheology.^14^

Superhydrophobic surfaces exhibit a diverse range of macro- and microscale applications. These include drag reduction for flow transport, protection against corrosion, icing, and biofouling, microflow manipulation and mixing, biological fluid synthesis and analysis, and so on.^9,15−22^ Considering such diverse ranges of applications, the fluid in contact with superhydrophobic surfaces may show both Newtonian and non-Newtonian rheology.^23−25^

In this Review, we attempt to discuss studies conducted to address the shear flow dynamics of Newtonian and non-Newtonian fluids over superhydrophobic surfaces, while focusing on the developed mathematical and numerical models. Our motivation for the current work originate from the lack of an up-to-date review focusing on the developed mathematical and numerical methods, despite remarkable evolutions of such modeling approaches. In addition, our intention is to highlight recent works on non-Newtonian flows over superhydrophobic surfaces, while emphasizing on the importance of developing new research as well as possible opportunities.

In what follows, we first introduce the main concepts that are frequently used in the present Review, followed by detailing the existing and potential applications of superhydrophobic surfaces. We then provide a brief background of studies conducted to address Newtonian and non-Newtonian shear flows over superhydrophobic surfaces. Finally, we present a summary, discussing the evolution of the mathematical and numerical methods used to address this problem, followed by a critical discussion on the modeling challenges and a highlight of the new areas of research in this domain.

## Fluid Rheology

Fluid rheology can affect the flow dynamics over slippery surfaces.^26−28^ Unlike Newtonian fluids, the viscosity of non-Newtonian fluids is a function of the magnitude of the flow strain rate..^29,30^ Since slippery wall conditions influence the strain rate tensor, the flow viscosity field and, hence, the whole non-Newtonian flow dynamics is affected as well.^6,31^ More complex slippery conditions, e.g., the superhydrophobicity, cause more complex dynamics for the non-Newtonian flows,^23,32−36^ i.e., a feature that we will discuss further in this Review.

### Shear-Thinning Fluids

Shear-thinning behavior is exhibited by materials whose viscosity decreases under shear strain.^37,38^ Such rheology usually excludes yield stress values and other non-Newtonian behaviors, e.g., time-dependent effects, and it is commonly modeled by the power-law or Carreau models, which correlate the stress and strain rate tensors via a nonlinear apparent viscosity.^37−39^

#### Power-Law Model

**Figure 2:**
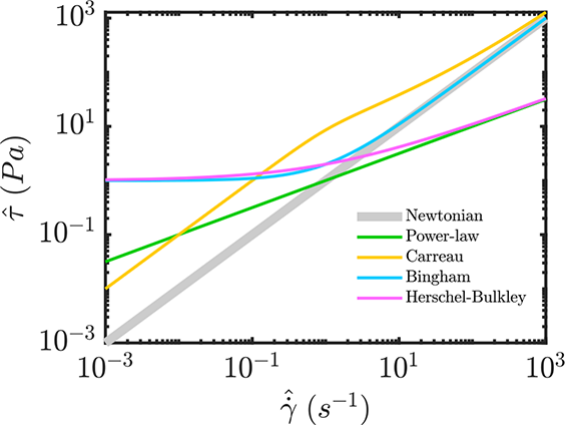
Schematic curves of the stress  versus strain-rate  for different rheological models.

The constitutive equation for a power-law shear-thinning fluid is defined as^37,38^
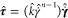
1where **τ̂** and  are the stress and the strain rate tensor, respectively. The norm (magnitude) of the strain rate tensor is defined as . In addition, *k̂* represents the consistency coefficient while *n* is the power law index. For a shear-thinning power-law material, *n* < 1 leads to a decrease in the apparent viscosity  when the norm of the strain rate grows. An example of the stress versus strain-rate curve for a purely shear flow of power-law fluid is shown in Figure 2.

#### Carreau Model

The apparent viscosity based on the Carreau model is defined as below:^38,39^
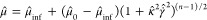
2where  and  are the viscosity at zero and infinite shear rate, respectively. In addition, κ̂ is a characteristic time coefficient and *n* < 1 represents the power law index for shear-thinning fluids. For the Carreau fluid flows, a Carreau number is usually defined representing a characteristic shear rate of the flow. As shown in Figure 2, a Carreau fluid indicates large viscosities at small shear rates. With an increase in the shear rate, the viscosity decreases.

### Yield Stress Materials

Yield stress materials are a branch of non-Newtonian fluids for which a threshold for the applied stress, called the *yield stress*, must be applied to elicit flow. When the applied stress is below the yield stress value, the material behaves like a rigid solid, forming the unyielded plug zones (including the stagnant zones); on the other hand, for applied stresses larger than the yield stress, the material deforms like a viscous fluid, forming the yielded zones. The boundary between the unyielded plug zone and the yielded zone is called the *yield surface*.^29,40^

The *viscoplastic* terminology may also be used to refer to *yield stress* materials. In this context, the viscoplastic terminology is commonly used by the fluid dynamicists, while the experimentalists mostly prefer the yield stress terminology.^40^ As discussed in detail in,^41^ it is also believed that the term *viscoplastic* can refer typically to the materials that show only viscous and plastic behaviors, while the term *yield stress* may be better used to refer to those that exhibit additional properties, e.g., elasticity, thixotropy and so on. However, for the purpose of this Review, we use the yield stress and viscoplastic fluid terminologies interchangeably.

Numerous fluids exhibit yield stress behavior in a variety of applications. Some common examples are waxy crude oil transported through a pipeline, molten polymers used in polymer extrusion and coextrusion processes,^42,43^ and foamed cement and cement slurry employed in the cementing of oil and gas wells.^44,45^ Additionally, numerous cosmetic products, including moisturizing creams and hair gel exhibit viscoplastic behavior.^40,46^ Several food products, including chocolate cream, butter, and jam, also show yield stress rheology.^40^ It may be interesting to note that for various biofluids, including human blood and serum albumin, and mucus, yield stress values are measured.^40,47^

#### Herschel–Bulkley Model

The most inclusive, yet simple, and widely used model that addresses many of the complexities of a viscoplastic non-Newtonian fluid, such as the yield stress, shear-thinning or shear-thickening and viscous effects, is the Herschel–Bulkley model, given as follows:^40^
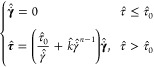
3where  is the fluid yield stress and  represents the norm of deviatoric stress tensor. The definitions of the other parameters used in the Herschel-Bulkley model are similar to those presented of the shear-thinning power law fluid. As illustrated in Figure 2, when the shear rate goes to zero, the Herschel-Bulkley fluid shows a yield stress value. On the other hand, for the large shear rate values, the Herschel-Bulkley fluid behavior mimics that of the power-law fluid.

#### Bingham Model

When the viscoplastic material only shows yield stress values, i.e., with no shear-thinning and shear-thickening effects (*n* = 1), the Herschel–Bulkley equation simplifies to the Bingham constitutive equation:^40^
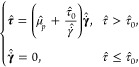
4where  is the plastic viscosity. In viscoplastic fluid flow problems, the Bingham number (*B*) is a crucial dimensionless parameter representing the ratio of the yield stress  to a characteristic stress, which for the shear flows of Bingham fluids may be defined as
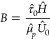
5where  and Ĥ are the characteristic velocity and length scales, respectively. Depending on the constitutive model under consideration (e.g., the Herschel-Bulkley or Bingham model), the Bingham number relation may change, which is due to the definition of the characteristic stress. For a Herschel-Bulkley material, the Bingham number can be defined as . Figure 2 shows that the Bingham and Hershel-Bulkley fluid behaviors mimic each other at the small shear rate values; however, as the shear rate increases the Bingham fluid behavior tends toward that of the Newtonian fluid.

## Slip Phenomenon

### Basic Concepts

**Figure 3:**
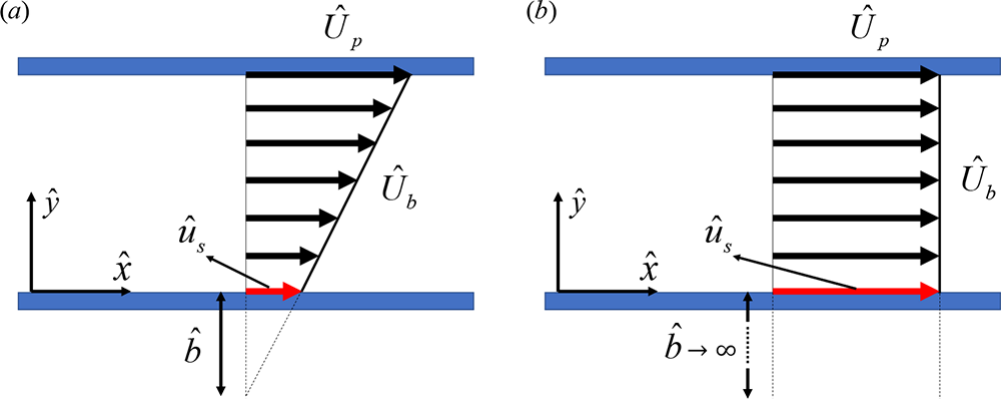
Couette flow of a Newtonian fluid with slip at the lower wall. The slip length, *b̂*, is visualized by an imaginary distance extrapolated from the wall to reach the no-slip condition. (a) Partial shear (*b̂* is nonzero and finite) and (b) no-shear  conditions. In the illustrated case, the upper wall moves with the velocity  and the lower wall is stationary.

The no-slip boundary condition at the (rigid) solid–fluid interface remains the prevailing and unquestioned orthopraxy in many fluid dynamics problems. However, during the past quarter century, a violation of the no-slip boundary assumption for the flow of Newtonian and non-Newtonian materials/fluids near the solid walls has been demonstrated through a large number of experiments.^12,48−55^ The phenomenon of slip refers to any situation at which the flow tangential velocity is different from that of the wall immediately at the contact zone.^51^ Understanding the slip phenomenon is of raising interest due to its practical applications in microfluidics,^56,57^ porous media,^58−60^ biological processes,^61,62^ electro-osmotic flows,^63,64^ extrusion through dies,^42^ lubrication,^65,66^ and sedimentation.^67^ Today, the standard linear boundary condition introduced by Navier^68^ is still used as a basis to study the slip phenomenon:
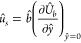
6where  is the fluid velocity at the wall,  is the velocity of the flow, *ŷ* is the coordinate axis perpendicular to the wall, and *b̂* is the slip length. For pure shear flows, *b̂* can be interpreted as an imaginary distance extrapolated from the wall, in order to satisfy the no-slip boundary condition (see Figure 3).

Let us review some of the important terms regarding the slip boundary condition. *Molecular slip (intrinsic slip)* takes place in a situation where hydrodynamics forces liquid molecules to slip against the solid molecules. It is believed that molecular slip occurs when intermolecular interactions are balanced with viscous forces. For molecular slip to begin, large values of shear rate must be exerted on the flow.^51^*Apparent slip* occurs due to the existence of a wall layer showing a small length scale between the base flow and the wall. In this case, the small scale layer satisfies the no-slip boundary condition; however, the base flow shows slip at the interface with the small scale layer.^51^ Apparent slip is observable in electrokinetics,^69^ acoustic streaming^70^ and flow over a gas layer.^71^ It is prevalent for non-Newtonian fluids to exhibit apparent slip, such as polymer solutions.^42,52,54,72−74^ When molecular or apparent slip is estimated by averaging an appropriate measurement over the experimental length scale, the reported value represents an *effective slip*.^51^

### Hydrophilic, Hydrophobic, and Superhydrophobic Surfaces

**Figure 4:**
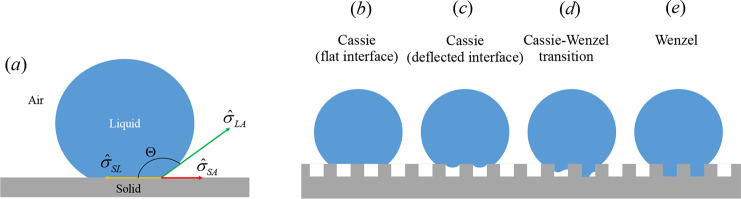
Water drop on a surface where the surface tensions and the contact angle are illustrated (panel a) and different states of the liquid drop on a textured surface (panels b–e).

Based on how a surface interacts or sticks to water drops, the surface can be categorized as hydrophilic (e.g., absorbing a drop) or hydrophobic (e.g., repelling a drop). When a surface shows a high surface energy, e.g., glass, a water drop tends to wet the surface. In contrast, a water drop beads up on a surface with low surface energy, e.g., Teflon. The contact angle is the prevalent choice in characterizing the wetting property of a surface wet by liquid/water. Young’s equation of static (equilibrium) contact angle is widely used to relate the contact angle with the surface tensions:^75^
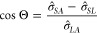
7where Θ is the Young’s contact angle, and , , and  are the surface tensions, i.e., energy per unit surface, of the solid/air, solid/liquid and liquid/air interfaces, respectively (see panel a of Figure 4). Surfaces with Θ < 90° are considered as hydrophilic while those with Θ ≥ 90° are known as hydrophobic.^9,25^ The presence of surface roughness or chemical heterogeneities causes a range of values for the equilibrium contact angle between the advancing (Θ_*A*_) and receding (Θ_*R*_) contact angles. Such a nonuniqueness of the equilibrium contact angle is referred as the contact angle hysteresis.^25^

Inspired by the lotus leaf, the superhydrophobic surfaces have been created based on adding micro-nano scale protrusions on the hydrophobic surfaces in order to decrease the surface wettability and, hence, improve the slippery condition.^10,11^ Micro-nano groovy structures are a type of well-known protrusions experimentally created on the hydrophobic/hydrophilic surfaces. The purpose is to trap air in the formed cavities between the protrusions. The trapped air decreases the liquid/solid contact and induces the slip condition at its contact with the flowing fluid.^9,12^ The case where the liquid sits upon asperities and the trapped air pockets are bounded in between the surface and liquid is called the *Cassie* state (see panels b and c of Figure 4). However, in some conditions, the trapped air escapes and the liquid penetrates into the cavities and forms the *Wenzel* state (see panel e of Figure 4). There are various potential possibilities for the liquid/air interface in this context, which are outlined as follows. (i) The interface is assumed to remain nearly flat while pinned at the groove edges (i.e., forming the ideal Cassie state)^5,76,77^ (panel b of Figure 4). (ii) While the interface is pinned at the groove edges, it may deform toward the groove or the main flow^78,79^ (panel c of Figure 4). (iii) The liquid may partially fill inside of the groove, thus, forming a liquid/air interface depinned from the groove edges while in contact with the side and bottom walls of the groove^80−82^ (panel d of Figure 4). The occurrence of these situations depend on several factors, i.e., the system pressure, surface tension, and fluid’s rheology,^14,80,81,83,84^ to name a few.

Superhydrophobic surfaces are associated with large contact angles and low contact angle hysteresis, making a water drop on these surfaces unstable and sensitive to even small perturbations.^25,85^ The large contact angle of a water drop on a superhydrophobic surface causes rolling motion instead of sliding, which is associated with the elevated center of the drop mass well above the surface.^25,85^ This rolling drop motion causes removal of dirt and dust from the surface providing a unique feature for superhydrophobic surfaces, i.e., self-cleaning ability.^25,85,86^ Considering the micronano scale protrusions as the major difference between the superhydrophobic and hydrophobic surfaces, i.e., not the chemistry, the developed synthetic superhydrophobic surfaces are capable of showing contact angles approaching Θ ≈ 180° while having negligible hysteresis.^25,85,87^

Considering the Cassie state, the hydrophobicity of a surface can help in the stabilization of the formed liquid/air interface, by preventing the liquid penetration into the cavity. The equilibrium contact angle for the Cassie state (Θ_*C*_) could be related to that for the hydrophobic surface (i.e., Θ) as^25,85,88^
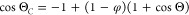
8where φ is the liquid/air interface fraction of the superhydrophobic surface. On the other hand, for the Wenzel state (Θ_*W*_), the hydrophobicity may be improved by the surface roughness:^25,85,89^
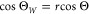
9where *r* represents the ratio of the actual wetted area to the projected area of the surface.

In addition to the increase in the contact angle, the presence of a liquid/air interface reduces the contact angle hysteresis; thus, the superhydrophobicity could be associated with the Cassie state.^88^ However, to maintain the Cassie state, the liquid/air interface fraction is limited to the system pressure, i.e., the pressure of the overlying flow near the interface. According to the Young’s law while assuming a liquid/air interface with a single radius of curvature, one simply finds:^25,90^
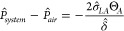
10where δ̂ is the width of the liquid/air interface and *P̂* is the pressure. As shown in Equation 10, an increase in the width of the liquid/air interface reduces the maximum system pressure at the onset of a liquid penetration to the cavity (i.e., at Θ_*A*_), pleading in favor of the Wenzel state formation. Therefore, there should be a balance between the enhanced hydrophobicity gained by the increase in the liquid/air interface fraction and the maximum pressure difference that is tolerable by the interface to avoid the Cassie to Wenzel transition.^25,90^

Before we end this section, it is also worth briefly introducing another class of biomimetic surfaces called the *liquid-infused* surfaces, which are inspired by pitcher plants of the genus Nepenthes.^91^ Liquid-infused surfaces are generated by infusing lubricant liquids with low surface tensions into the cavities of micro structured surfaces. This leads to formation of composite liquid–solid surfaces with slippery properties.^91,92^ The infused lubricant is immiscible with the working outer liquids in contact with the liquid-infused surface. Comparing with superhydrophobic surfaces, the liquid-infused surfaces show smaller values of effective slip; however, the liquid/lubricant interface is remarkably more stable than the liquid/air interface (of a superhydrophobic surface).^91,92^

## Applications of Superhydrophobic Surfaces

The flow over superhydrophobic groovy surfaces, experiencing the Cassie state, has many practical applications, e.g., in drag reduction, flow manipulation and handling, preventing biofouling layer formation, enhancing anti-icing and anticorrosion surface properties, etc.^9,15−22^ A drag reduction for laminar flows is usually challenging especially at micrometer-sized pipes with no-slip boundary condition.^93^ Surface modification using micro (nano) textures have been successfully used to reduce hydrodynamic friction in laminar and turbulent flows.^93^ The microgrooved surfaces are a type of superhydrophobic surfaces that promote trapping of air in the cavities and are capable of creating a shear-free liquid/air interface. Several studies^94−97^ have quantified the slip length and drag reduction on surfaces with trapped air pockets for laminar pipe and channel flows. Slippery superhydrophobic surfaces are not only of great interest for drag reduction applications but also for other types of applications such as heat and ion transport.^98,99^

In microfluidic devices, the objective is to manipulate small volumes of fluid through microscale conduits which exhibit huge hydrodynamic resistance in pressure-driven flows.^16^ A solution to this problem is the usage of smooth hydrophobic surfaces in order to decrease the surface resistance. The slip length for these hydrophobic surfaces is not more than tens of nanometers.^100,101^ Consequently, it is not possible to gain special advantage of such hydrophobic surfaces for the pressure-driven microfluidics applications.^16^ Thanks to the trapped gas in their surface cavities, superhydrophobic surfaces exhibit slip lengths of the order of several microns.^102,103^

Bahga et al.^104^ have analytically studied the electro-osmotic flows over weakly charged, groovy superhydrophobic surfaces. The authors have concluded that the electro-osmotic mobility tensor is calculable based on the slip length and charge profiles. The electro-osmosis flow over anisotropic groovy superhydrophobic surfaces has been also studied by Belyaev and Vinogradova.^105^ The authors have theoretically described the electro-osmotic flow over groovy surfaces and derived a relation between the hydrodynamic slip length and electro-osmotic mobility tensors. The large effective slip length of textured superhydrophobic surfaces has been exploited to prevent the clogging or adhesion of suspended analytes in microfluidic devices.^106^ Inducing significant transverse flow, patterned slippery surfaces have been used to enhance the mixing efficiency of Newtonian and power-law fluids in the microfluidic devices.^107,108^

While in this section, the general applications of the superhydrophobic groovy surfaces were brought and discussed, in the next two sections, the specific cases of non-Newtonian fluids (including yield stress and shear-thinning rheology) used in those applications will be addressed.

### Drag Reduction

Transport of non-Newtonian fluids, i.e., typically showing yield stress and shear-thinning behaviors, is of high industrial significance.^109−112^ This process is observed in the petroleum, pulp and paper, and food processing industries, as well as industries dealing with transport of polymeric fluids and mined slurries, pharmaceutical and cosmetic industries, and so on, in which the working materials typically show yield stress and shear-thinning rheology beside other complex rheological features.^109,112,113^ Several studies for the flow of non-Newtonian fluids through pipes have been conducted, emphasizing the practical importance of such flows. Early on, Metzner and Reed^114^ established correlations for frictional pressure losses using a range of experimental data for non-Newtonian fluids, including yield stress and shear-thinning rheology. Similar approach was developed for the yield stress fluids by Hanks and Pratt.^115^ In the petroleum industry, it is commonplace to perform non-Newtonian pipe flow experiments for enhancing the accuracy of hydraulic predictions and understanding the hydrodynamics of new fluids that are being pumped.^116^ In mining industry, viscoplastic shear-thinning models are usually used to model homogeneous slurries and many experimental efforts for capturing the transitional flow regimes have been conducted to quantify the flow physics.^117^

Reducing the skin friction between the flowing fluid and the wall has been an effective scenario to facilitate the transport of fluids through ducts and pipes, in both laminar and turbulent flows.^118,119^ The drag reduction can be obtained by the use of superhydrophobic surfaces on which a layer of trapped gas isolate the direct contact of fluid and the solid wall.^119−123^ A decrease in friction or wall shear stress facilitates the transport of laminar flows and it also delays transition to turbulence.^124^ In addition, within the turbulent flow regime, the superhydrophobic wall surfaces can also provide drag reduction.^119,122^ In addition to the above approach, in some other studies, surfaces with riblet topology or roughness, which can be produced via groovy structures, are used in order to decrease the skin friction.^95,96,125,126^

Based on the above-mentioned brief discussion, it is concluded that superhydrophobic surfaces can be utilized for drag reduction purposes through pipes and ducts. Such pipes/ducts are widely used to transport different types of fluids, such as yield stress and shear-thinning fluids, in many practical applications.^8,22^ This drag reduction can be related to the effective slip length of superhydrophobic groovy structured surfaces.^15,16,102^ Here, the interaction and coupling between the fluid rheology and the superhydrophobic surface topology are of high interest and significance.

### Flow through Microfluidic Devices

For many applications, the rheology of fluid flowing in microfluidic devices shows yield stress and/or shear-thinning characteristic. A case in point is the giant electrorheological fluid (GERF) .^127,128^ Electrorheological fluids (ERFs), composed of dielectric particles suspending in insulating oil, are a type of fluid known as *smart* colloid, showing tunable viscosity under the effect of an external electric field.^127^ Therefore, an external electric field can change the viscosity of ERF by a few orders of magnitude. A sufficiently strong electric field causes a solidification of ERF into an anisotropic solid with a yield stress. In other words, the GERF fluid shows yield stress behavior under the applied electric field. By the use of GERF, scientists have developed a series of fully chip-embedded soft-valves and made a significant progress in fluidic-based automatic droplet control systems. GERFs can have a yield stress up to 300 kPa in response to an electric field, providing the possibility to digitally control the microvalve.^127^

There are several other couplings between microfluidics and complex fluids. Flow of polymer solutions (typically showing yield stress and shear-thinning rheology) through microscale restrictions shows new instabilities.^129^ New rheometers have been designed by the use of microfluidic technology.^129^ Nghe et al.^129^ have studied different configurations of complex fluids including polymer breakup in microfluidic systems, polymer solution flows close to the microchannel wall, shear banding flows in microchannels, and flow of concentrated solutions of microgel particles in microchannels. In some cases, the yield stress behavior, such as a formation of a plug zone has been observed (e.g., the flow of concentrated solutions of microgel particles, and shear banding flow) .^129^ There are many other studies dealing with yield stress and shear-thinning fluids in microfluidic systems for a variety of applications, such as electrokinetic flow of yield stress fluids,^130,131^ drop formation of Carbopol solutions,^132^ biological fluids manipulations,^133^ and magnetic-field induced yield stress flows.^134^

Many biological fluids show complex non-Newtonian rheology.^135^ Globular proteins, e.g., serum albumins (which are among the constituent proteins in mammalian blood and are known to strongly affect the microstructure of biofluids) cause yield-like behavior for a wide range of concentrations.^135−138^ Subsequently, blood also shows yield stress values beside shear-thinning features, which provide necessary rheological properties for its physiological functions.^47,135^ Globular proteins are also known as vital constituents of many foods, as well as pharmaceutical and cosmetic products.^135^ Blood and its components, e.g., globular proteins and plasma solutions, and pharmaceutical materials are among prevalent fluids synthesized in microfluidic devices for several purposes, e.g., disease diagnosis, and drug development and delivery.^24,139^ In this context, recent studies have revealed remarkable advantages of using superhydrophobic surfaces in design and development of microfluidics systems. This includes applications in protein adsorption,^140,141^ selective deposition of molecules and cells in diagnostic applications,^24,142^ blood compatibility in diagnostic platforms and prosthetic grafts,^143,144^ clinical surgery,^145^ and effective drug delivery.^139,146^

## Newtonian Fluids

In this section, the major studies and methods developed to describe the flow dynamics of Newtonian fluids over superhydrophobic surfaces are presented and discussed. First, the techniques used to conduct the mathematical modelings for the slip length and velocity field are presented. The corresponding numerical studies and methods are discussed, afterward.

### Mathematical Modelings

**Figure 5:**
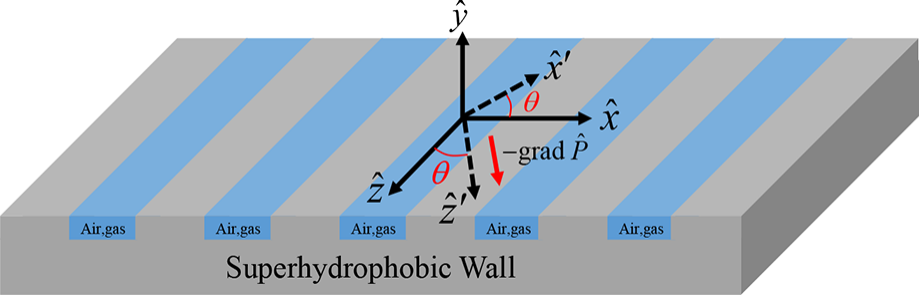
Schematic of an oblique flow configuration (0 < θ < 90°) on a superhydrophobic groovy surface.

The modeling of the slip dynamics of Newtonian fluid flows over superhydrophobic groovy surfaces has been usually considered for two configurations, i.e., longitudinal and transverse flows; in these cases, the applied pressure gradient makes the angle of θ = 0 and θ = 90° with the groove direction, respectively. An oblique flow configuration, on the other hand, concerns the case where 0 < θ < 90°, as schematically presented in Figure 5.

#### Creeping Flows

Typically, the effective slip lengths for the longitudinal and transverse flow configurations are symbolized by  and , respectively. For a creeping flow, assuming no-shear liquid/air interface, for simple one-dimensional textures, the relation between longitudinal and transverse effective slip lengths is obtained as .^18^

For a flat liquid/gas interface at a no-shear condition (i.e., the ideal Cassie state with no meniscus curvature), several studies on pressure driven flows^147−149^ have revealed the following relation for the effective slip length:
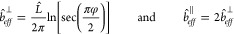
11where *L̂* is the groove period and φ represents the fraction of the liquid/air interface.

To predict the local slip length  at the liquid/air interface, a model called the *gas cushion model* has been proposed by Vinogradova:^150^
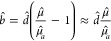
12where *d̂* is the depth of the air (gas) layer and μ̂ and  are the viscosities of liquid and air (gas), respectively.

Some of the main mathematical techniques reported in the literature used for modeling of Newtonian flows over superhydrophobic surfaces have been based on the theories of perturbation and superposition.^148,151,152^ As one of the pioneering studies, Lauga and Stone^148^ have quantified the effective slip in the Hagen–Poiseuille Stokes flows, while considering arrays of no-slip and no-shear condition positioned periodically on the pipe wall. The Stokes equations have been solved based on perturbing the no-slip Hagen–Poiseuille flow. Their results have shown a good agreement with the experimental measurement.

Asmolov et al.^153^ have developed an effective slip length model for shear-driven flows of Newtonian fluids over weakly slipping rectangular stripes. In their work, the no-slip boundary condition is used for the solid–liquid interface at the wall, and the local slip length, b̂, is quantified the slip condition at the interface between trapped gas in the striped surface and the liquid. In other words, the liquid/gas interface is assumed to obey the ideal Cassie state. The local slip length, *b̂*, is considered to be small, in comparison with the scale of the heterogeneities, i.e., *L̂*. Considering the slip velocity as a perturbation velocity on the superhydrophobic wall, the authors have perturbed the Stokes equation, i.e., , and reached a Laplace’s equation for the perturbation velocity, i.e., Δ*û* = 0, where  is the base flow velocity, *û* is the perturbation velocity,*P̂* represents the system pressure, and Δ*a*nd ∇ are the Laplacian and gradient operators, respectively. The slip boundary condition on the liquid/air interface has been modeled by the linear Navier slip law. Assuming a periodic flow transverse to the groove direction (e.g., in the *x̂* direction according to Figure 5), a cosine Fourier series form is considered to be the solution of the perturbation velocity. Finally, by applying the no-slip and slip boundary conditions on the superhydrophobic wall, the coefficients of the Fourier series are calculated, and later used to find both longitudinal and transverse slip velocities and lengths. Taking into account the leading and second order asymptotic terms, the modeled slip lengths address the slip singularity at the edge of stripes.

The longitudinal and transverse slip length are modeled as^153^
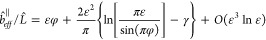
13
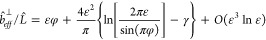
14where , , and γ = 0.5772157 is Euler’s constant. Here, δ̂ is the width of the liquid/air interface and L̂ is the groove period (i.e., the scale of the heterogeneities). The above solution, which is developed for a free shear flow, equivalent to an infinitely thick channel, allows solving for the conditions where the slip length is small, i.e. before reaching the no-shear condition (which is characterized with very large slip lengths) that is the case in eq 11.

**Figure 6:**
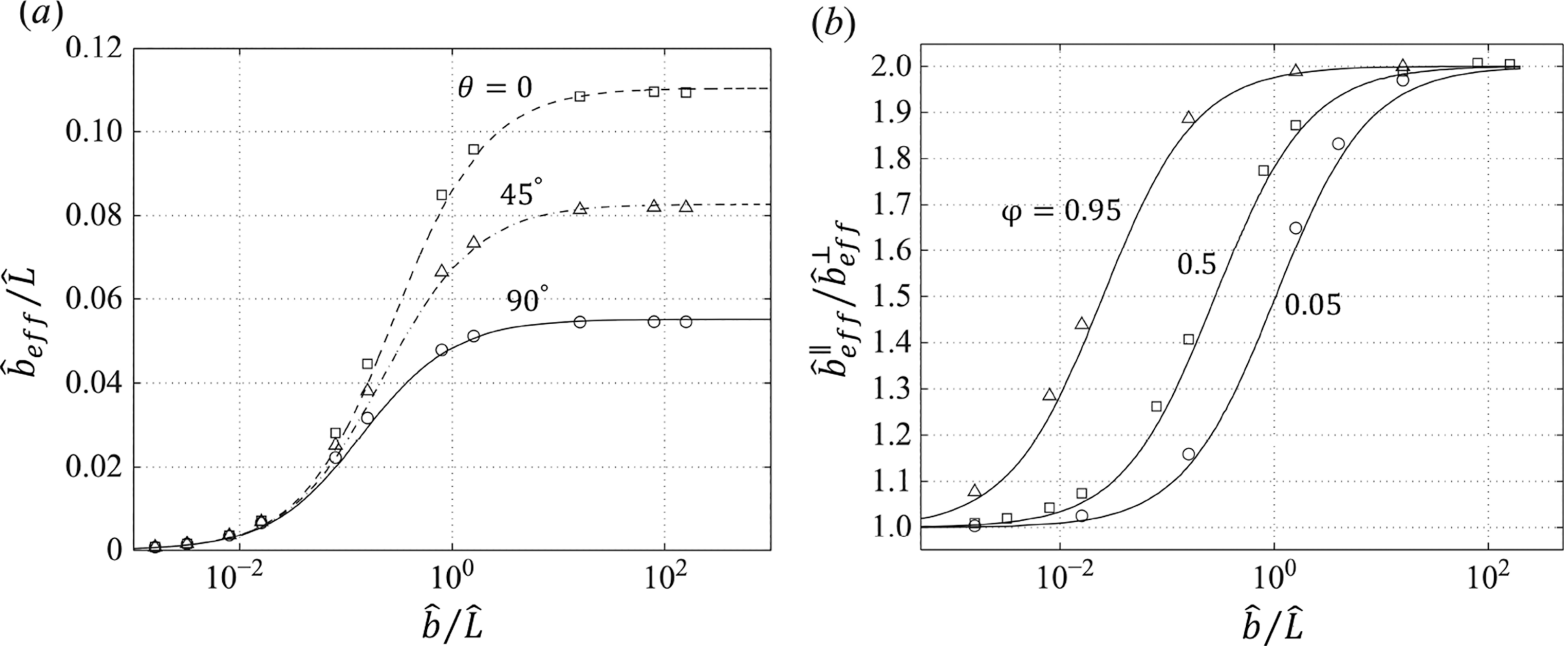
(a) Normalized effective slip length for longitudinal (θ = 0), transverse (θ = 90°), and oblique (θ = 45°) flows with φ = 0.5. (b) Ratio between the longitudinal and transverse effective slip lengths versus the normalized local slip length. Symbols show the simulation data while curves illustrate theoretical results. Adapted from Belyaev and Vinogradova^16^ with permission. Copyright 2010 Cambridge University Press.

Belyaev and Vinogradova^16^ presented an effective slip length model for the pressure-driven flow of Newtonian fluids over rectangular stripes. A Poiseuille channel flow was considered where the lower wall had striped structures showing an effective slip length, and the no-slip condition was assumed for the upper wall. Similar to the study of Asmolov et al.,^153^ for the longitudinal effective slip modeling, the Laplace equation for the perturbation velocity was solved with the use of Fourier series expansion. However, for the transverse slip modeling, due to stream-wise dependency of pressure gradient, the Navier–Stokes equation was written based on stream function  and vorticity , i.e., . The Poisson and Laplace equations for the perturbations of the stream function and vorticity, i.e.,  and Δω̂ = 0, were solved by a similar method to that of the longitudinal slip case. In both longitudinal and transverse slip cases, the applied no-slip and slip boundary conditions led to a dual series problem, which was resolved using a technique introduced by Sneddon.^154^ The formulas for the longitudinal and transverse slip length were obtained as
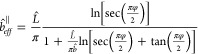
15
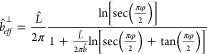
16where eqs 15 and 16 are valid for any small, intermediate, and large value of the slip length , while converging to eq 11 when *b̂* increases. As shown in Figure 6, for a smaller θ and a larger φ, the effective slip length is larger. The effective slip length converges when the local slip length becomes sufficiently large, e.g., when ,  as demonstrated by eq 11.

**Figure 7:**
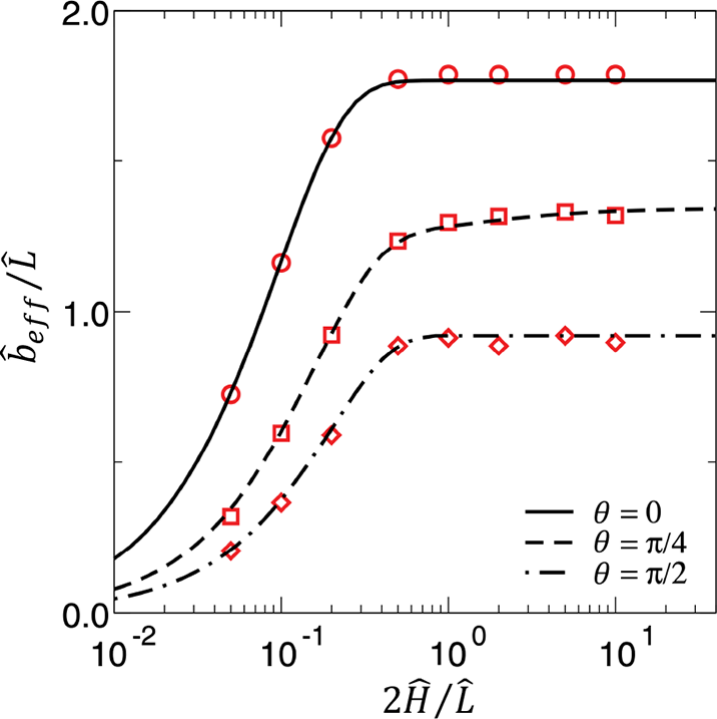
(a) Normalized effective slip length versus the normalized channel height. Here, φ = 0.75 and . Symbols show the simulation data while curves illustrate theoretical results. Adapted from Schmieschek et al.^155^ with permission. Copyright 2012 American Physical Society.

Schmieschek et al.^155^ generalized the tensorial effective slip theory, initially developed for thick channels, to any channel thickness. Subsequently, the obtained eigenvalues of the effective slip length tensor were dependent on the channel gap thickness and surface local slip properties. The upper wall was assumed to satisfy the no-slip condition; however, the lower wall was a superhydrophobic groovy surface with a varying local slip length. Similar to the previous studies,^16,151^ the authors^155^ considered the Stokes equation and the Navier slip law as governing equations. Since the equations were linear, the velocity profile was considered to be the superposition of velocities of a no-slip parabolic Poiseuille flow  and a superimposed perturbation velocity . For the case of longitudinal stripes, the Laplace equation is the governing equation for the perturbation velocity, for which the solution for the slip-induced velocity, û, has a Fourier series form. Applying the no-slip and slip boundary conditions, the Fourier series form of the solution yields a trigonometric dual series, describing the complete physics of flow hydrodynamic and effective slip in the longitudinal direction.^155^ The obtained dual series can be solved numerically; however, in the case of thin and thick channels, there also exist exact solutions. The authors have conducted almost the same procedure to solve for the transverse slip, for which the Poisson’s equation of the perturbation stream function is the governing equation. Based on their generalization formula, the previously obtained effective slip models in the literature for the asymptotic cases of thin and thick channel were retrieved. As shown in Figure 7, the effective slip length was larger for a thicker channel (i.e., larger Ĥ), while showing a converging trend for . Based on Figure 7, for , which represents a large local slip length, when the channel is sufficiently thick, e.g., , one can realize that , which is consistent with the previous findings in the literature, e.g., Belyaev and Vinogradova.^16^

Wang^156^ developed an analytical solution to calculate the slip length for the flow of Newtonian fluids over a surface with parallel grooves. The idea was to consider the flow field close to the surface as a noninertial flow, leading to neglecting the advective terms in the Navier–Stokes equations. Consequently, the Navier–Stokes equations shrinked to a simpler Laplace’s equation that has a series solution. Two velocity fields were considered: the first region was located inside the grooves and the second region was close to the surface and outside the grooves. To derive the final solution, the series solutions for both regions (obtained based on eigenfunction expansions) were matched at the interface between the regions, i.e., the liquid/gas interface. The authors calculated the slip length for the longitudinal and transverse groove orientations and concluded that the slip length was different for these configurations, implying the anisotropic behavior of the flow.

Developing a scaling approach, Ybert et al.^157^ formulated the slip length based on the generic surface characteristics, such as roughness length scale, depth and solid fraction of the interface. As an example of their scaling, the authors scaled the flow shear rate with an imposed plug-like flow velocity close to the surface and the width of the liquid/solid interface . For the asymptotic case of a zero liquid/solid interface, the effective slip length was obtained as .

Bazant and Vinogradova developed the tensorial form of the effective slip length for Newtonian flows over superhydrophobic surfaces,^158^ which allowed solving the oblique flow scenario (0 < θ < 90°), based on the solutions of the longitudinal and transverse flow configurations using an *a priori* known rotation tensor. For a Newtonian flow, the effective slip length tensor  for any configuration of the pressure gradient and the groove directions can be calculated using the following relation:^16,158^
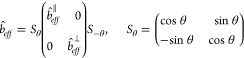
17where *S* is the transform matrix (or rotation tensor) and θ represents the angle between the directions of the pressure gradient and the groove.

The effective slip length was quantified for a thin channel limit and any flow configuration (i.e., longitudinal, transverse and oblique) by Feuillebois et al.,^159^ demonstrating the largest slip occurring in the longitudinal configuration. Back in 2011, Vinogradova et al.^151^ provided a review on their work on Newtonian flows over superhydrophobic surfaces, from thick to thin channels and from longitudinal to transverse flows.

**Figure 8:**
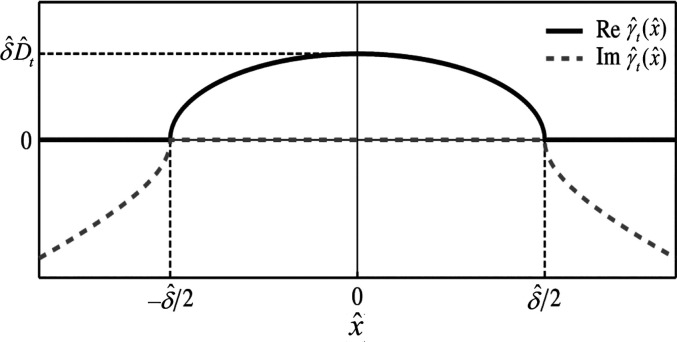
Real (solid line) and imaginary (dashed line) part of . Adapted from Schönecker and Hardt^161^ with permission. Copyright 2013 Cambridge University Press.

In the above-mentioned works, the liquid/air interface was modeled using a constant local slip length. Having the no-slip condition at the liquid/solid contact means that a step-like distribution was assumed for the local slip length on the superhydrophobic surface. On the other hand, as discussed by Schönecker et al.,^160^ it would be more physically relevant to consider an smooth distribution for the local slip length. This was achieved by considering a constant shear stress condition at the interface.^160,161^ In an attempt to find the distribution of the local slip length, Schönecker and Hardt^161^ considered a cavity flow problem, representing one shallow groove, to be able to make a connection between the flow dynamics inside the cavity and the local slip length. Employing the lubrication theory, they found an elliptical distribution for the local slip length that agreed well with the numerical results. For transverse flows, they found *b̂* = *Nγ̂*_*t*_, where *N* is the viscosity ratio between the inner and outer fluids, and γ̂_*t*_ is the slip length function described as (see Figure 8):
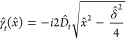
18where  is a variable that quantifies the maximum of the elliptic slip length distribution, δ̂ is width of the liquid/fluid interface, and x̂ represents the axis toward the transverse flow with its origin at the middle of the interface. As shown in Figure 8, the real part of  determines the physical distribution of the local slip length.

In the work of Schönecker et al.,^160^ the obtained elliptical distribution for the local slip length was used to address the Newtonian flow over microstructured surfaces with an enclosed fluid inside the micro grooves. Using the Goursat theory, the authors developed a solution which was a superposition of the plain Couette flow and Philip’s^162^ solution. Within the framework of the developed solution, the outer flow dynamics could find functionality with respect to the width of the liquid/fluid interface and viscosity ratios between the inner and outer fluids.

**Figure 9:**
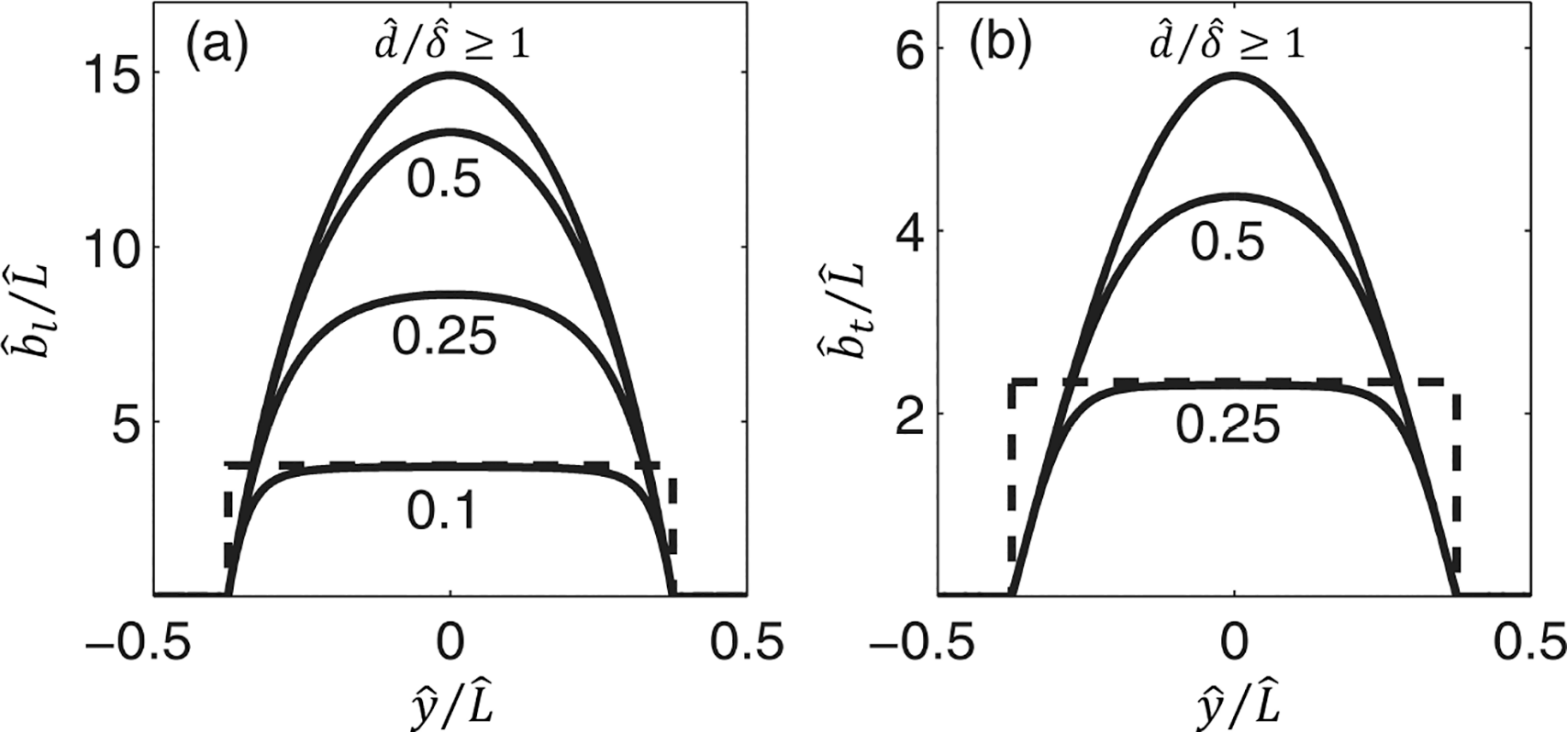
Example of the longitudinal (a) and transverse (b) smooth local slip lengths. The dashed line shows the constant local slip length prediction. *L̂* is the groove period, *d̂* represents the depth of groove, while δ̂ is its width. *ŷ* is the axis normal to the groove direction. Adapted from Nizkaya et al.^163^ with permission. Copyright 2014 American Physical Society.

Nizkaya et al.^163^ generalized the gas cushion model, by considering the flow dynamics inside the cavity of a superhydrophobic surface, leading to obtaining smooth distributions for the local slip lengths of the longitudinal and transverse flows (see Figure 9). The developed models for the local slip length showed functionality with the viscosity contrast between the liquid and the enclosed gas as well as the geometry of the cavity. They reported a generally larger local slip length for a larger depth over width of the cavity. Interestingly, regardless of the values of the viscosity contrast, the cavity aspect ratio or even the liquid/air interface fraction, for relatively deep grooves the profiles of the local slip length converged to a single curve. Dubov et al.^164^ revisited the hydrodynamics of Newtonian flows over longitudinal superhydrophobic surfaces with arbitrary shaped grooves. The authors focused on the local slip condition at the liquid/gas interface, assuming a large viscosity contrast between the liquid and gas. Their model correlated the eigenvalues of the local slip tensor to the texture parameters, e.g., the width and local depth of the groove. It was demonstrated that, for the deep grooves, the eigenvalues of the local slip length did not depend on the groove depth, i.e., they only depended on the viscosity ratio, groove width and an angle representing the groove shape. On the other hand, for shallow grooves, the eigenvalues were strongly affected by the local depth of the groove.

**Figure 10:**
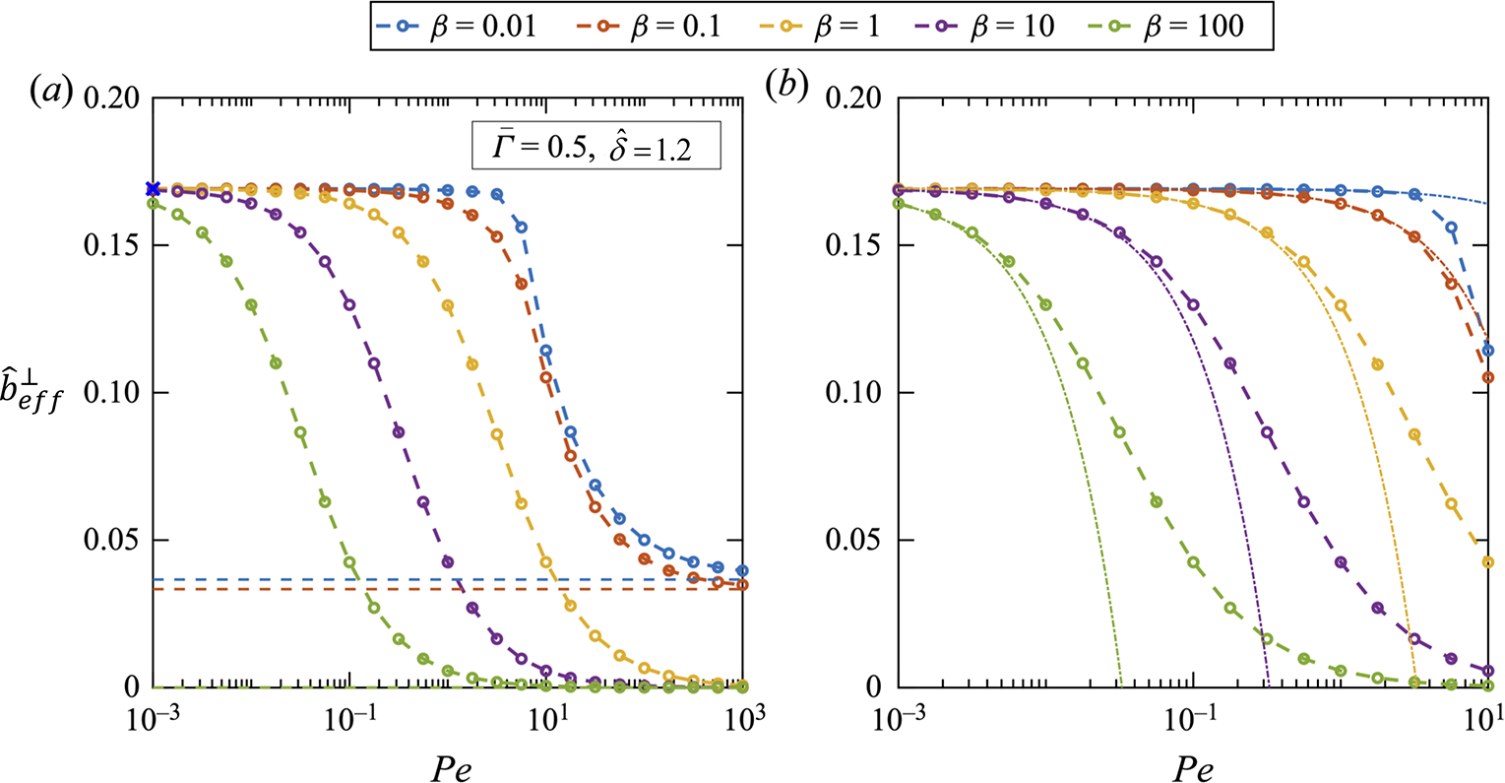
Effective slip length as a function of Peclet number (*Pe*; representing inverse of the surfactant diffusion on the meniscus) for different strengths of Marangoni stress (represented by β) at surfactant concentration Γ̅ = 0.5 and meniscus width δ̂ = 1.2. In panel a, the horizontal dashed line corresponds to *Pe* → *∞* and β → 0. Panel b is a refined view of panel a. The dashed lines with circle show the numerical data while the dotted lines represent a perturbation solution when *Pe* → 0. Adapted from Mayer and Crowdy^165^ with permission. Copyright 2022 Cambridge University Press.

Assuming a transverse Stokes flow with a flat meniscus, Mayer and Crowdy^165^ studied the effects of insoluble surfactant on immobilization of superhydrophobic surfaces. Perturbation theory, which was employed for the small surface Peclet and Marangoni numbers, was combined with numerical solutions to gain an understanding regarding the role played by Peclet and Marangoni numbers and the surfactant load. As shown in Figure 10, an increase in Peclet and Marangoni numbers leads to a decrease in the effective slip length, e.g. immobilizing the superhydrophobic surface at *Pe* = 10 and β = 100. Peaudecerf et al.^166^ also demonstrated the reduced drag reduction of superhydrophobic surfaces due to the traces of surfactants, by developing careful simulations and measurements. The authors showed that even at low surfactant concentrations, the surfactant-induced stresses were significant, leading to immobilization of the superhydrophobic surface. In another work, Temprano-Coleto et al.^167^ studied the impairment of superhydrophobic drag reduction due to presence of surfactant, through theoretical, numerical and experimental works. Based on their theoretical model, the authors demonstrated that the grating length, i.e., the ratio between the width of the liquid/air interface and the half-channel height (here φ), was the key parameter in predicting the ratio between the actual and surfactant-free (clean) slip; i.e. . The key finding was that the surfactant effects could be predicted by a single parameter, i.e. the ratio between the grating length and a mobilization length. More recently, Tomlinson et al.^168^ solved the three-dimensional problem of surfactant-contaminated flows in superhydrophobic channels. The developed model allowed addressing the complex flow system dynamics, i.e., the competition between Marangoni effects, bulk and interfacial diffusion and advection, shear dispersion, and the exchange of surfactant between the bulk and the interface. Explicit closed-form approximations of drag reduction were derived via mapping out the asymptotic regions. The developed one-dimensional model results agreed well with those of the full three-dimensional numerical simulations in the literature.

Longitudinal flows through superhydrophobic annular pipes have been theoretically studied by Crowdy.^169^ One wall was decorated by a pattern of no-shear stripes and the other was a fully no-slip boundary. It was found that, for pipes with the inner no-shear stripes, there is an optimal ratio of the inner-outer pipe radii corresponding to the maximum effective slip, which was approximately in the range of 0.5–0.6. Such optimal pipes depended only weakly on the no-shear surface patterning. Due to the negative effects of boundary point singularities on the generated slip, the author suggested maximizing the size of uninterrupted no-shear regions instead of having smaller no-shear regions for a superhydrophobic wall with a fixed no-shear area. Most recently, Zimmermann and Schönecker^170^ analytically solved the pressure-driven Stokes flow through superhydrophobic and liquid-infused tubes and annular pipes, while assuming finite local slip length or shear stress along the rotationally symmetrical longitudinal slits. The presented solution allowed assessment of the viscosity effects of the air or the liquid that filled the slits beside the evaluation of the role played by the microgeometry of the slits.

**Figure 11:**
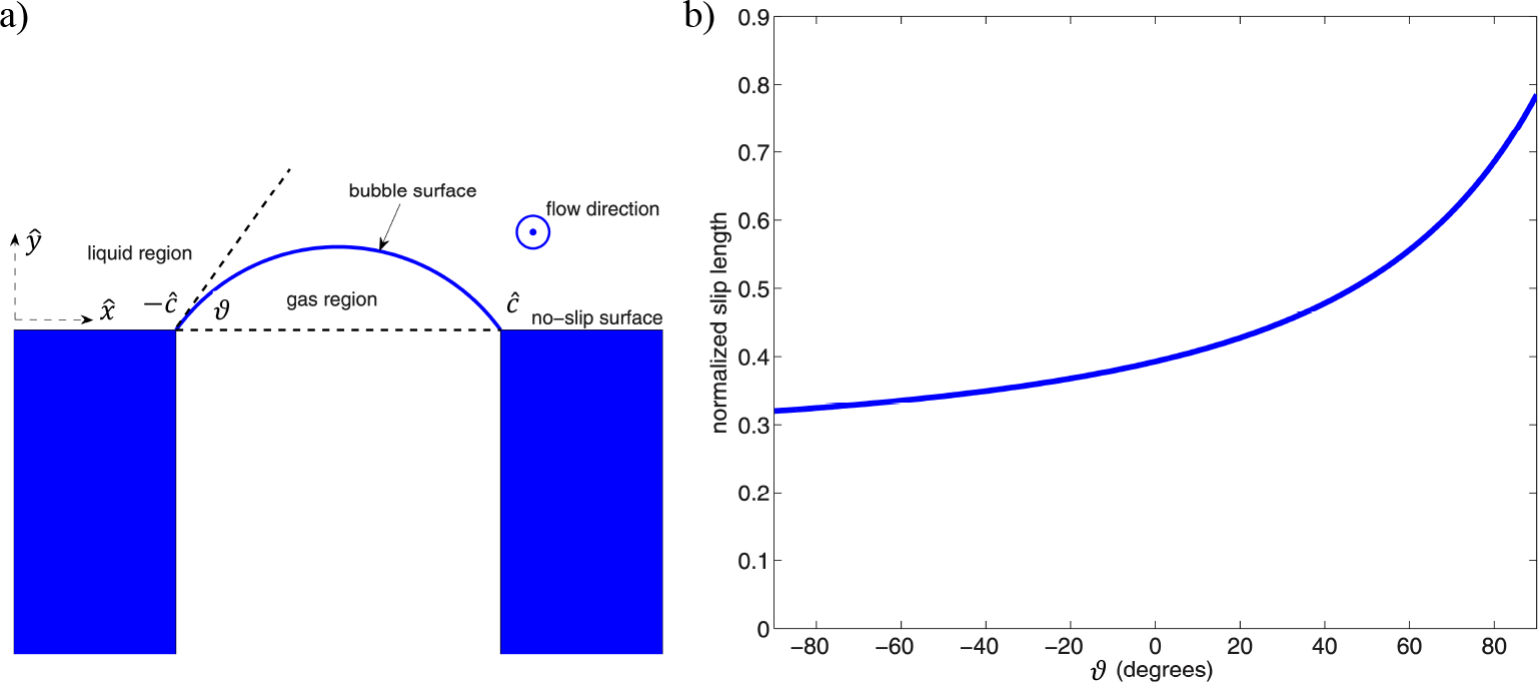
Schematic of (a) the longitudinal flow with a deflected meniscus and (b) the normalized slip length versus the meniscus angle. Adapted from Crowdy^173^ with permission. Copyright 2010 AIP Publishing.

In almost all the aforementioned studies, the interface of liquid/air (or liquid/fluid in general) was assumed to obey the ideal Cassie state; i.e., the interface was flat while pinned at the groove edges. Sbragaglia and Prosperetti^171^ attempted to address the longitudinal flow over a groovy superhydrophobic surface with a deflected liquid/air interface. The authors assumed that the interface was pinned at the groove edges and it was slightly deflected. They employed a perturbation analysis to solve the problem and concluded that the competition between the variation in the cross-sectional area of the flow passage and the change in the velocity field determined whether the effective slip length increased or decreased. Davis and Lauga^172^ developed an analytical solution to address the transverse shear flow over a bubble mattress. The no-shear and no-slip conditions were assumed on the bubble surface and the liquid/solid interface, respectively. A conformal mapping was used to transform the Laplace equation for the streamfunction to a toroidal coordinate system. The authors demonstrated an increase in the friction when the bubble was sufficiently curved and protruded into the shear flow, which was in line with the experimental and numerical results. Crowdy^173^ introduced an analytical solution for the longitudinal shear flow over a bubble mattress through employing a sequence of conformal mappings. The author demonstrated an increase in the effective slip length when the bubble shape changes from a concave (protruded into the surface) to a convex (protruded into the flow) shape (see Figure 11). In addition, it was shown that the calculated effective slip lengths were generally larger for the longitudinal flow compared to the transverse one. Crowdy^174^ extended their work beyond the dilute limit to develop a more accurate solution for the effective slip length over a bubble mattress for longitudinal flow configuration. The developed solution is accurate for a much larger range of no-shear fractions. A new perturbation analysis was developed by Crowdy,^175^ to address the subphase gas and meniscus curvature effects, for longitudinal flows over superhydrophobic surfaces. Assuming a weak deflection of the meniscus and a large viscosity contrast between the entrapped subphase gas and the working fluid, integral expressions for the first-order correction to the effective slip length were developed. This work extended the earlier work by Sbragaglia and Prosperetti,^171^ leading to a new integral expression for the first order slip length correction. Crowdy^176^ developed a solution for the longitudinal flow over a superhydrophobic grating where the menisci partially invaded the cavities while weakly deflected. The author, first, analytically solved the problem for a flat meniscus depinned from the top of grating and displaced into the cavity, leading to calculation of the slip length. Then, the developed solution was combined with an integral identity that provided a first-order correction of the slip length for the deflected meniscus.

**Figure 12:**
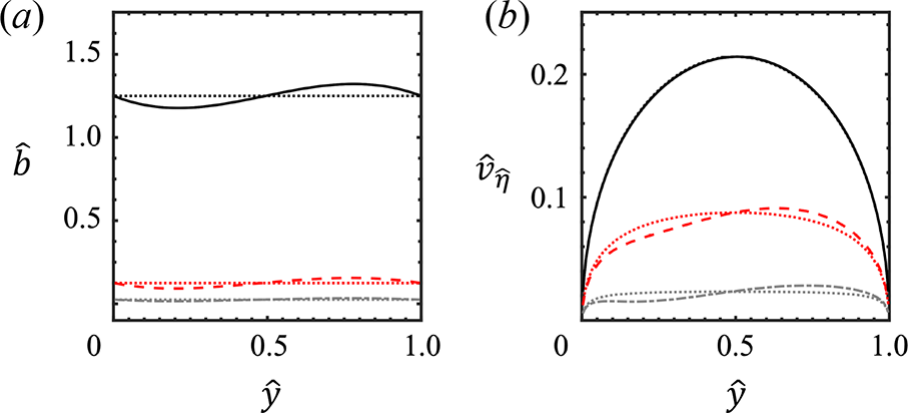
(a) Slip length distribution and (b) slip velocity profile in *ŷ*. Data are calculated at *d̂* = 0.1 and Ca = 0.3 while the solid, dashed and dash-dotted curves represent the results for μ̂ = 0.02, 0.2, and 1, respectively. Dotted lines show the results for a flat meniscus. Adapted from Asmolov et al.^178^ with permission. Copyright 2020 Cambridge University Press.

Marshall^177^ derived an exact solution for the longitudinal flow in channels with symmetrically aligned groovy superhydrophobic surfaces, by using conformal mappings and loxodromic function theory. The developed solution addressed the flow dynamics for both flat and weakly deflected liquid/gas interfaces, by introducing contour integrals of functions of a partial derivative of the working fluid’s velocity field corresponding to the flat interface. Asmolov et al.^178^ quantified the collapse of lubricant-infused surfaces due to depinning of the meniscus from the groove edge, by considering the capillary effects, the liquid/lubricant viscosity ratio, and the groove aspect ratio. To develop the model, the authors correlated an outer shear flow with the inner flow inside the groove, assuming that the lubrication theory was valid and the inner flow showed a parabolic velocity profile of zero flow rate (i.e., the lubricant flow mimics a lid-driven cavity flow with a zero flow rate). The authors concluded that the liquid/lubricant interface could collapse and depin from the front edge; however, it first touched the bottom wall before such a depinning for very shallow grooves. Profiles of the local slip length and the slip velocity are presented in Figure 12 while compared with those of a flat interface. For a smaller lubricant viscosity, the local slip length and slip velocity are larger. The effect of slowly varying meniscus curvature was studied on the inertial internal flows by Game et al.^78^ The problem was addressed by approximating the meniscus shapes as circular arcs. In addition, near the inlet, it was considered that the meniscus protrudes into the groove while away from the inlet where the liquid pressure decreases the meniscus is drawn into the main flow. A hybrid analytical-numerical method was developed to solve the nonlinear three-dimensional problem as a sequence of two-dimensional linear problems. It was shown that the inertial effects could significantly reduce the flow rate when the pressure difference across the microchannel was constrained by the advancing contact angle of the liquid and the surface tension.

#### Inertial Flows

**Figure 13:**
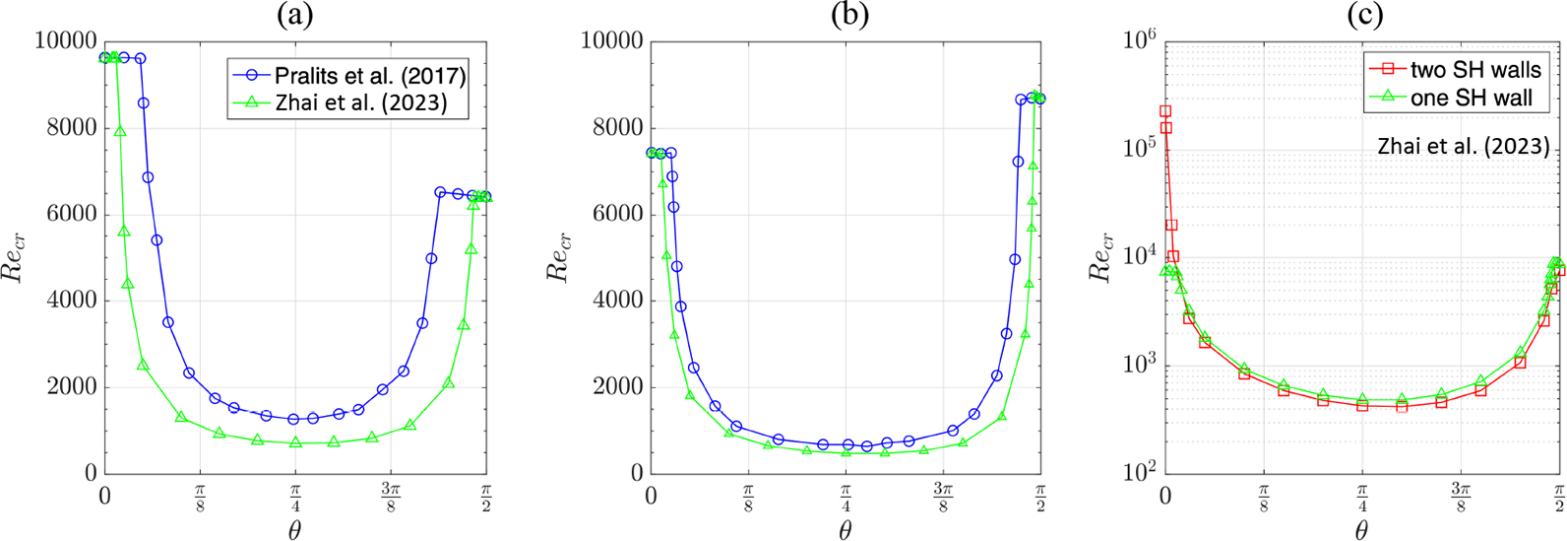
(a,b) Critical Reynolds number as a function of groove orientation angle for the channel with one superhydrophobic wall; comparison between the results of Pralits et al.^180^ and Zhai et al.^181^ (c) Critical Reynolds number calculated by Zhai et al.^181^ for channels with one and two superhydrophobic walls. Here, it is assumed that *b*_*eff*_^∥^/*b*_*eff*_^⊥^ = 2, while for (a) *b*_*eff*_^∥^ = 0.07 and (b,c) *b*_*eff*_^∥^ = 0.155. Adapted from Zhai et al.^181^ with permission. Copyright 2023 American Physical Society.

A linear stability analysis of the plane Poiseuille flow in channels with longitudinal grooves was studied by Yu et al.^179^ A shear-free flat liquid/air interface was assumed and the effects of the width of this interface and the groove period on the stability picture was explored. A BiGlobal linear stability analysis via the pseudospectral method revealed both stabilizing and destabilizing effects of the microstructured surface on the flow, predominantly depending on the ratio of the groove period over the channel height (i.e., ∝ *l*). For small values of *l*, i.e., corresponding to thick channels, stabilizing effects were observed, which was in line with the results of a local stability analysis assuming a homogeneous wall slip condition. On the other hand, new modes of instabilities were found at lower critical Reynolds numbers when *l* increased (i.e., for thinner channels). Modal and nonmodal linear stability analysis of the Poiseuille flow in channels with one or two microgrooved surfaces (with oriented groove directions with respect to the pressure gradient direction) were performed by Pralits et al.^180^ It was shown that the Squire’s theorem was not valid for the assumed flow, although the Squire modes were always damped. For a channel with one superhydrophobic wall, the authors reported the appearance of a streamwise wall-vortex mode at very low Reynolds numbers when the grooves were sufficiently oriented. While Pralits et al.^180^ found the flow with one superhydrophobic wall more unstable than that with two superhydrophobic walls, Zhai et al.^181^ reported the lowest value of the critical Reynolds number for the flow with two superhydrophobic walls (see Figure 13). In addition, Zhai et al.^181^ reported an increase in the critical Reynolds number as the difference between the tilt angles (groove orientation angles) of the two superhydrophobic walls increased. Tomlinson et al.^76^ conducted linear stability analyses for lid- and pressure-driven flows in superhydrophobic groovy channels, with a longitudinal groove direction. Assuming flat liquid/air interfaces, the results of the global stability analyses revealed new modes of instabilities for both types of the flow at small critical Reynolds numbers.

A brief summary of the mathematical efforts conducted for the shear flows of Newtonian fluids over superhydrophobic surfaces is presented through Table 1.

**Table 1:** Summary of the Mathematical Modeling Efforts Conducted for Newtonian Flows^a^

Ref. no.	Flow regime/type	Macro/micro geometry	Interface/B.C.	Key finding
(147), (162)	Creeping/(Longitudinal, Transverse)	(Plate, Channel)/Slot	Flat/No-shear	Velocity field for the first time
(148)	Creeping/(Longitudinal, Transverse)	Pipe/Stripe	Pipe arc/No-shear	Effective slip length
(16)	Creeping/(Longitudinal, Transverse)	Channel/Groove	Flat/Local slip length	Effective slip length for thick channels
(155)	Creeping/(Longitudinal, Transverse)	Channel/Groove	Flat/Local slip length	Effective slip length for thick to thin channels
(158)	Creeping/Oblique	(Plate, Channel)/Groove	Flat/Local slip length	Tensorial slip for oblique configuration
(160), (161)	Creeping/(Longitudinal, Transverse)	Plate/Groove	Flat/Constant shear stress	Distribution and functionality of local slip length
(163)	Creeping/(Longitudinal, Transverse)	Plate/Groove	Flat/Local slip length	Distribution and functionality of local slip length
(165)	Creeping/Transverse	Plate/Groove	Flat/No-shear	Surfactant induced reduction of effective slip length
(167)	Creeping/–	Channel/Grating	Flat/Marangoni shear	Surfactant effects were predicted by a single parameter
(169)	Creeping/Longitudinal	Pipe/Stripe	Pipe arc/No-shear	Velocity field and effective slip length
(170)	Creeping/Longitudinal	Pipe/Slit	Pipe arc/Local slip length	Slip dynamics for annular pipe with inner filling fluid
(173), (174)	Creeping/Longitudinal	Plate/Bubble mattress	circular arc/No-shear	Effective slip length for deflected interface
(176)	Creeping/Longitudinal	Plate/Grating	Deflected/No-shear	Effective slip length with partially filled cavities
(178)	Creeping/Transverse	Plate/Groove	Deflected/Local slip length	Interface shape and local slip distribution
(76)	Inertial/Longitudinal	(Cavity, Channel)/Groove	Flat/No-shear	New superhydrophobic wall-induced unstable modes
(180), (181)	Inertial/Oblique	Channel/Groove	Flat/Local slip length	Most unstable modes occurred at intermediate values of θ

a Macro/Micro geometry: Geometry of the bulk flow and that of the superhydrophobic surface. B.C.: boundary condition at the liquid/air interface.

### Numerical Simulations

In this section, the numerical methods used to simulate fluid flows over superhydrophobic groovy surfaces are presented and discussed. There are four main modeling approaches in the literature, including the finite element/volume, molecular dynamics simulation, dissipative particle dynamics, and lattice Boltzmann methods. Except for the finite element/volume method, the other methods are known to be particle-based techniques.

In finite element/volume methods the computational domain is discretized to two/three-dimensional elements for which the Navier–Stokes equations are solved.^182^ These methods are suitable for macroscale flow simulations. In molecular dynamics method, the molecules are modeled by spherical particles interacting with one other based on the Lennard-Jones potentials.^183^ In dissipative particle dynamics and lattice Boltzmann methods, a finite element of fluid representing a large number of molecules are modeled.^184,185^ These models are used to perform mesoscopic simulations as they have access to larger (smaller) time and length scales compared to molecular dynamics (finite element/volume) simulations.

#### Creeping Flows

Using the commercial computational fluid dynamics code Fluent, Davies et al.^186^ performed numerical simulations of a flow through microchannels with superhydrophobic walls having transverse ribs. The authors used the first order upwind scheme to discretize the advective terms and the SIMPLE algorithm to establish the velocity-pressure coupling. Assuming a flat meniscus, a significant decrease in the frictional pressure drop was reported, compared to classical Poiseuille flow with smooth walls. The authors showed that an increase in the width of liquid/air interface (with a shear-free condition) and a decrease in the channel hydraulic diameter led to a reduction in the pressure drop and a growth in the effective slip velocity.

A channel flow with superhydrophobic walls was modeled using computational fluid dynamics package Fluent by Ou and Rothstein.^187^ The water/air interface was assumed to be flat (with no deflection) and the shear-free condition was considered for the interface. Considering a large surface tension, small width of the water/air contact, or low system pressure, the authors were allowed to assume a flat water/air interface in their simulations. The no-slip condition was considered at the water/wall contact. A maximum slip velocity of more than 60% of the average velocity was calculated at the middle of the shear-free meniscus. The authors found a good agreement between the results of numerical simulations and those of their μ-PIV experiments.

Priezjev et al.^188^ investigated the Couette flow of Newtonian fluids with a superhydrophobic patterned stationary wall. The authors performed simulations using both the finite element method, for solving the Navier-stokes equations, and the molecular dynamics simulation method, to quantify the effective slip length. They reported a good agreement between the results of the finite element and molecular dynamics simulation methods when the ratio of the slip region width to the molecular diameter was larger than 10. In this regime, the effective slip length grew monotonically with an increase in the width of the slip region (i.e., the flat meniscus) to a saturation value. For ratios smaller than 10 and transverse flows, the nonuniform interaction potential at the patterned surface caused a rough surface behavior, leading to a strong decrease in the effective slip length, i.e., smaller than the hydrodynamics predictions. On the other hand, for the longitudinal flow, due to a translational symmetry, the molecular scale roughness effects were eliminated and the reduced molecular ordering above the wetting regions caused an increase in the effective slip length, i.e., much larger than the hydrodynamic predictions. The authors reported a strong correlation between the effective slip length and the liquid structure of the first fluid layer near the patterned wall, i.e., highlighting the molecular ordering effects on the slip dynamics.

Teo and Khoo^189^ studied the meniscus curvature effects on the Couette and Poiseuille flows over superhydrophobic groovy surfaces in longitudinal configuration by conducting finite element numerical computations in Matlab. Considering a shear-free liquid/air interface, the effective slip length was shown to be strongly affected by the meniscus curvature. For large interface deflections toward the liquid phase, large shear-free fractions, and thinner channels, the effective slip length approached zero or became negative for the Poiseuille flow, which was associated with the significant flow blockage effects. Game et al.^190^ studied the longitudinal Poiseuille flows in superhydrophobic groovy channels by using Chebyshev collocation and domain decomposition methods. Using the natural boundaries provided by the solid–liquid–gas triple contact points, the authors first decomposed the computational domain into smaller subdomains. Each single subdomain was then mapped to a canonical rectangular domain, where the Chebyshev collocation method was used to solve the governing equations. The final solution was calculated through imposing continuity conditions between the solutions of neighboring domains. Based on the calculated numerical results, the authors suggested important principles to optimize the flow enhancement, i.e., constructing channels with fewer, wider grooves while minimizing the meniscus curvature. To this end, the authors concluded that the groove depth should be sufficiently small in order to maximize the area taken up by the liquid, while being sufficiently deep to minimize the role of the gas shear stresses.

**Figure 14:**
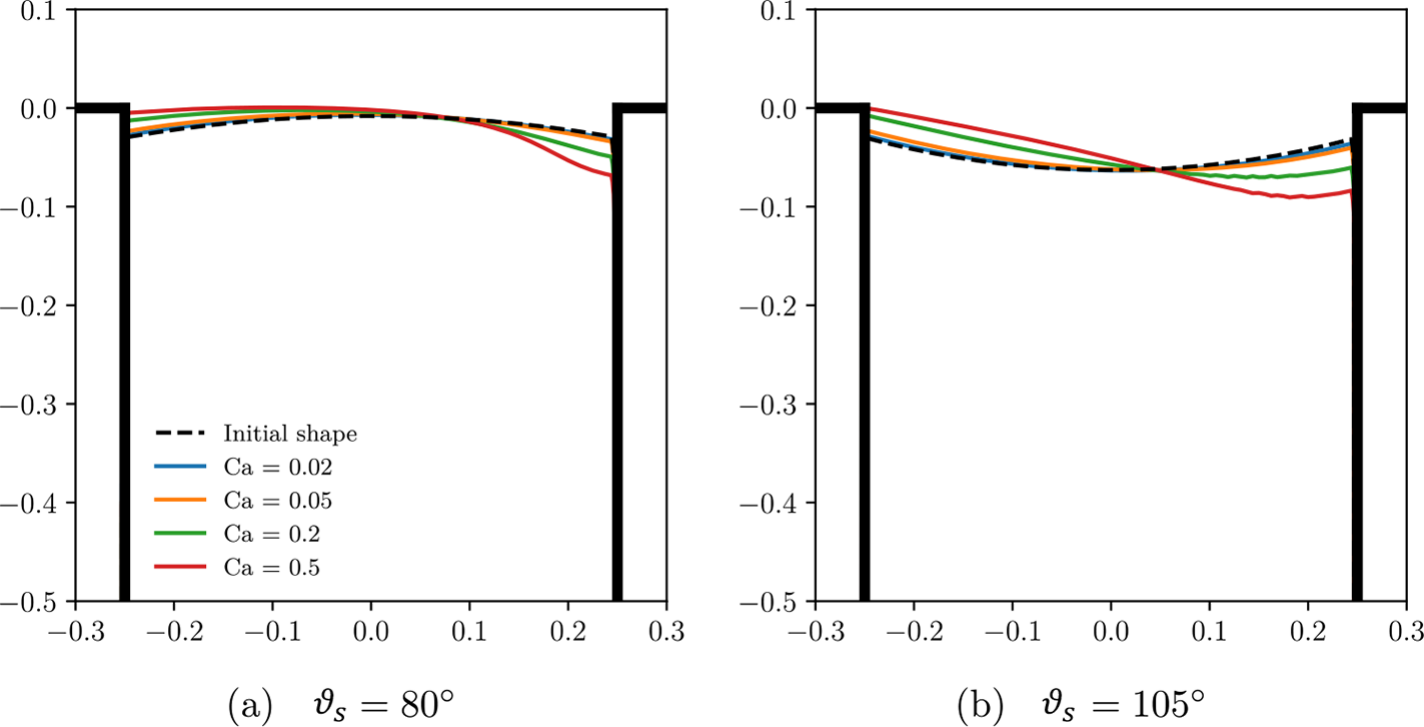
Snapshot of the meniscus shape at different Capillary numbers (Ca). Here, . Two different static contact angles (ϑ_*s*_; i.e., the angle between the groove wall and the meniscus) are considered for the initial shape. Adapted from Ge et al.^191^ with permission. Copyright 2018 American Physical Society.

Using a multiscale numerical framework, Ge et al.^191^ studied the shear flow of Newtonian fluids over lubricant-infused surfaces. The characteristic contact line velocities at liquid–solid interfaces were first calculated by phase field simulations. The extracted data were then used in microscale two-phase simulations to explore the shear and cavity flows dynamics. The authors found that the effective slip length was strongly affected by the filling fraction of the cavity (i.e., indicative of the normalized initial depth of the lubricant in the cavity). For an initial filling fraction of 0.94 and for small values of the Capillary number, the effective slip length was nearly not affected; however, when the Capillary number increased to 0.01 of the viscosity contrast between the two liquids, a possible drainage of the lubricant from the cavity was found (see the Capillary number effects on the meniscus shape in Figure 14). Gaddam et al.^192^ studied drag reduction of Newtonian flows in superhydrophobic microchannels with micro ridges of different shapes. Using a finite volume solver and considering the liquid/air interface evolution, the authors reported the maximum drag reduction for lotus-like micro ridges, when compared to that for square, narrow, rounded and circular ridges. They also reported a partial shear condition at the liquid/air interface.

Cottin-Bizonne et al.^103^ conducted molecular dynamics simulations of a pressure-driven fluid flow through microchannels for which the walls were decorated with narrow rectangular protrusions. The authors confirmed that the mesoscopic roughness caused by the protrusions greatly modifies the interfacial contact between the liquid and the solid wall, leading to enhance the slippage. In another interesting study, Cottin-Bizonne et al.^193^ performed molecular dynamics simulations of flows over a corrugated hydrophobic surface. The authors reported a pressure below the capillary pressure leading to a superhydrophobic state. Based on the continuum mechanics, they proposed a macroscopic estimate for the effective slip length, showing a good agreement with the results of molecular dynamics simulations. Molecular dynamics simulations of polymer liquid flows through channels with patterned walls were performed by Tretyakov and Muller.^194^ Their simulation results were compared with those of a channel with flat walls and significant differences were found for liquids in the Cassie state. Using Couette and Poisuelle flows, the hydrodynamic boundary position (i.e., where the flow shear stress and the wall friction are balanced) and the slip length were characterized. The authors reported that the hydrodynamic boundary position for almost all their simulations was located above the peak of the grooves (into the flow). Simulation results demonstrated that the slip length sensitively depended on the pressure. When the pressure was high, the slip length was found to be small while the friction coefficient was found to be high.

The dissipative particle dynamics method is a coarse-grained, momentum-conserving method used for mesoscale fluid analyses. Simulations of the flow near striped superhydrophobic surfaces were conducted by Asmolov et al.,^153^ with the use of a version of dissipative particle dynamics method without conservative interactions. The fluid/solid interaction on the superhydrophobic surface was modeled by a tunable-slip method, defining an effective friction force. The authors employed the open source package ESPResSo to perform their simulations. They used the results of simulations to verify the accuracy of their asymptotic estimation of the slip length. Asmolov et al. focused on the edge effects, associated with step-like discontinuities in the local slip length, and they concluded that such edge effects reduce the effective slip below the surface-averaged value and cause anisotropy. Zhou et al.^195^ performed dissipation particle dynamics simulations to evaluate a semianalytical solution that they had developed to characterize the effective slip length for the flow through a channel with a superhydrophobic groovy wall. In their analysis, the channel height was allowed to have arbitrary values. The authors reported a good agreement between the results obtained from the mesoscopic simulations and predictions of the semianalytical method.

**Figure 15:**
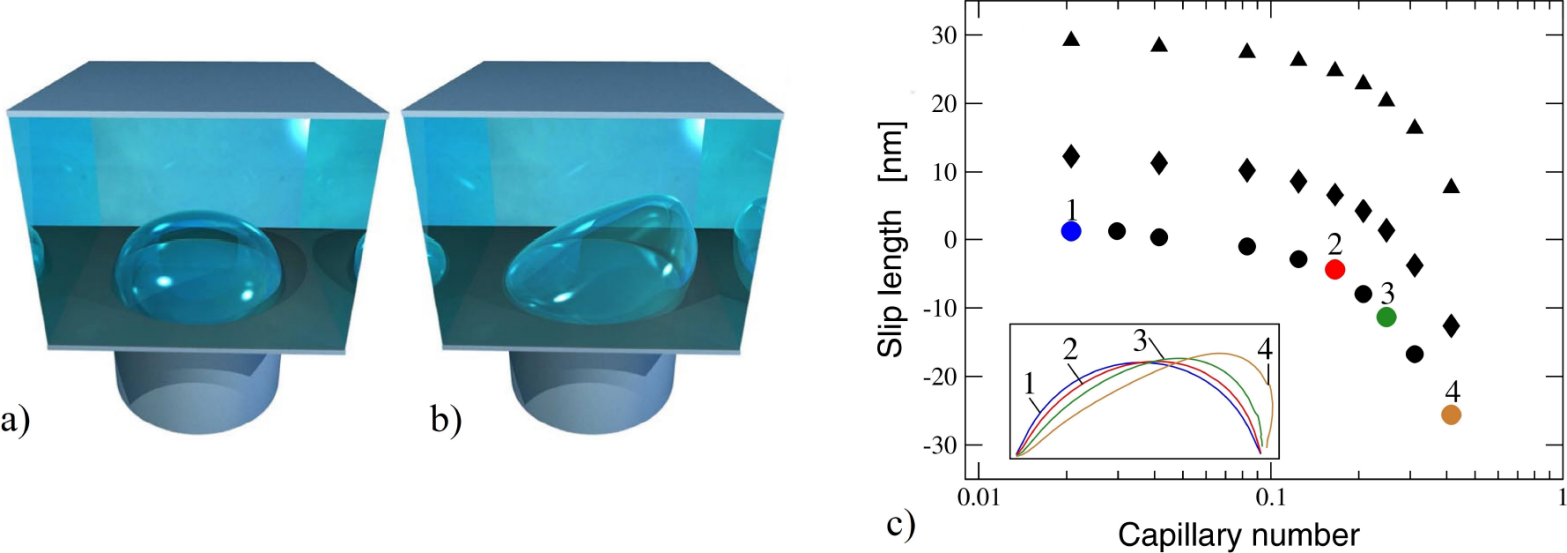
Snapshots of simulations of bubbles on structured surfaces: (a) Ca = 0.02 and (b) Ca = 0.4. (c) Slip length versus capillary number presented for three protrusion angles ϑ = 63°, 68°, and 71°, which are represented by the symbols from uppermost to lowermost, respectively. The liquid/gas interface is illustrated for four capillary numbers distinguished by the numbers from 1 to 4. Adapted from Hyvaluoma and Harting^196^ with permission. Copyright 2008 American Physical Society.

Hyvaluoma and Harting^196^ performed two-phase lattice Boltzmann simulations of a Couette flow over structured surfaces exhibiting attached gas bubbles. In the simulations, the bubbles were allowed to deform due to the viscous stresses. The authors reported a decrease in the effective slip with increasing the shear rate that contradicted with the results of previous experimental studies, implying the limitations of the experiments in accounting the bubble deformations. Two examples of their flow simulations showing the bubble deformation are depicted in Figure 15. In addition, the results for the relation between the slip length and capillary number are depicted in Figure 15. The capillary number is defined as Ca  where μ̂ is the fluid viscosity, *â* is radius of the holes, and  and  are the shear rate and the surface tension, respectively.

Benzi et al.^197^ developed a mesoscopic model for the fluid/wall interactions and suitably implemented the corresponding boundary conditions in a lattice Boltzmann equation of a single-phase fluid in a microchannel with heterogeneous slippery walls. Defining a slip function, the authors were able to observe similar trends to that of molecular dynamics simulations and experimental measurements for the slip length and velocity. Schmieschek et al.^155^ performed lattice Boltzmann simulations of the plane Poiseuille flow in channels with longitudinal and transverse grooves. The computations were based on a three-dimensional lattice, i.e., the D3Q19 model, and revealed a flow behavior that followed that predicted by the Navier–Stokes equation. Periodic boundary conditions were assumed in two directions, i.e., longitudinal and transverse directions, reducing the simulation domain to a two-dimensional system. A good agreement between the numerical model results and those of the analytical model were found. Dubov et al.^164^ developed a numerical framework to solve for the two-phase flow of Newtonian fluids over superhydrophobic groovy surfaces. The cavity flow inside the groove was solved using the D3Q19 lattice Boltzmann model, leading to calculating the gas flow velocity and shear gradient at the meniscus, eventually providing the eigenvalues of the local slip length. The calculated eigenvalues then specified the boundary conditions at the liquid/gas interface, used to develop a numerical solution for the outer Stokes flow through implementing the Fourier series expansions. The results of the developed numerical framework showed better approximation for the velocity at the meniscus.

#### Inertial Flows

**Figure 16:**
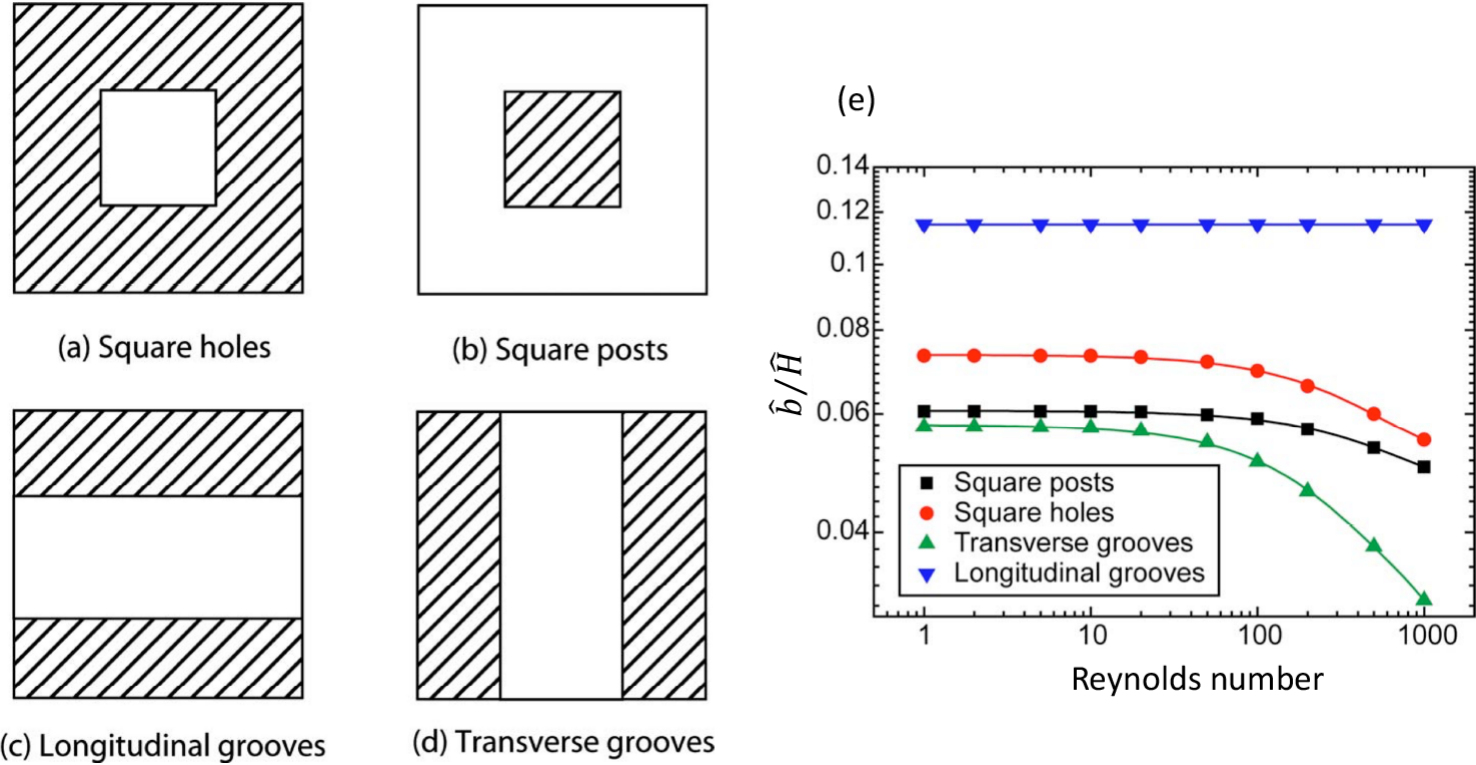
(a–d) Schematics of different types of microstructures on the superhydrophobic surface and (e) normalized slip length versus the Reynolds number. In panels a–d, the liquid/air interface is shown in white while the hatched area represents the liquid/solid contact. Adapted from Cheng et al.^198^ with permission. Copyright 2009 AIP Publishing.

Almost all the previously mentioned studies considered creeping shear flows over superhydrophobic and lubricant-infused surfaces. However, there have been efforts on addressing the inertial flow dynamics over such complex boundary conditions as well. A pressure-driven flows through channels with superhydrophobic surfaces patterned with longitudinal and transverse grooves, posts and holes were numerically studied by Cheng et al.^198^ The finite volume and finite element methods were combined to discretize the governing Navier–Stokes equations. Coupling between the pressure and velocity was established using the CLEARER algorithm.^199^ Considering flat liquid/air interfaces, the authors validated their numerical results for the case of a transverse flow with the existing analytical relations and they found a good agreement. The authors concluded that the channel confinement positively affected the effective slip length for channels with square posts and longitudinal grooves. In contrast, it negatively affected the effective slip length for channels with square holes and transverse grooves. As shown in Figure 16e, it was found that the Reynolds number did not affect the effective slip length for the longitudinal channels while it deteriorated the effective slip length of square posts, holes, and transverse grooves.

**Figure 17:**
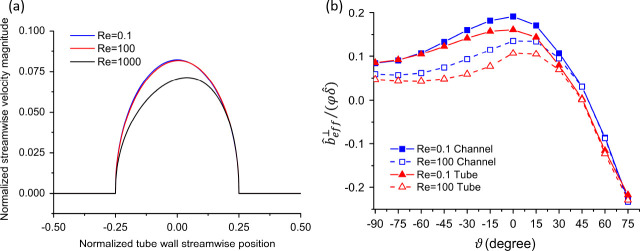
(a) Slip velocity profile (for φ = 0.5, *l* = 0.125, and ϑ = 0), and b) effective slip length (for φ = 0.5 and *l* = 4), at different Reynolds numbers. Adapted from Teo et al.^200^ with permission. Copyright 2014 Springer Nature.

Inertial nonturbulent flows of Newtonian fluids in microchannels and microtubes with transverse grooves and ribs were studied by Teo and Khoo,^200^ with a focus on the meniscus curvature effects. The authors showed that at low Reynolds numbers and large channel heights and pipe diameters, the critical protrusion angle (of the meniscus) corresponding to the zero effective slip length was about ϑ_*c*_ ≈ 62° – 65°, i.e., independent of the shear-free fraction and the flow geometry. A decrease in the channel height or pipe diameter for a given shear-free fraction led to a reduction in ϑ_*c*_. On the other hand, an increase in the Reynolds number caused a decrease in the slip velocity and the effective slip length and such an effect was more pronounced for the pipe flow compared to the channel flow (see Figure 17).

Conducting finite volume simulations, Ahuja et al.^201^ characterized the liquid/lubricant interface in a dovetail cavity, considering viscosity contrasts of 0.2–1 between the outer fluid and the lubricant, in the laminar range of 100 ≤ *R* ≤ 1000. The meniscus shape was characterized for the studied range of the viscosity contrast and Reynolds number. For a fixed lubricant, the smaller viscosity contrast and the lower Reynolds number led to a better retention of the lubricant. The authors reported three stable meniscus shapes, i.e. flat, concave and asymmetric meniscus, and two failure ones, i.e., partial and complete failure where the outer flow invades into the cavity partially and completely, respectively. In another work, Sharma et al.^202^ numerically studied the slip flow in microchannels with lubricant-infused surfaces decorated with holes and posts. Using a finite volume solver, simulations were conducted for the Reynolds numbers of 1 ≤ *R* ≤ 1000 and the viscosity contrasts of 0–1 between the lubricant and the outer fluid. The authors reported a decrease in the effective slip length with increase in the Reynolds number, which was more significant for surfaces with holes compared to those with posts at small viscosity contrasts.

A brief summary of the numerical efforts conducted for the shear flows of Newtonian fluids over superhydrophobic surfaces is presented through Table 2.

**Table 2:** Summary of the Numerical Modeling Efforts Conducted for Newtonian Flows^a^

Ref. no.	Flow regime/type	Macro/Micro geometry	Interface/B.C.	Key finding
(186)	Creeping/Transverse	Channel/Rib	Flat/No-shear	Growth of effective slip length for a larger φ and *l*
(187)	Creeping/Longitudinal	Channel/Stripe	Flat/No-shear	Good agreement with μ-PIV data
(188)	Creeping/(Longitudinal, Transverse)	Plate/Stripe	Flat/No-shear	Strong effects of molecular ordering on slip dynamics
(189)	Creeping/Longitudinal	(Plate, Channel)/Groove	Deflected/No-shear	Meniscus deflection strongly affected effective slip length
(190)	Creeping/Longitudinal	Channel/Groove	Deflected/Continuity	Principles for flow enhancement optimization
(191)	Creeping/Transverse	Plate/Groove	Deflected/Continuity	Quantified drainage of lubricant from cavity
(192)	Creeping/(Longitudinal, Transverse)	Channel/Ridge	Deflected/Continuity	Maximum drag reduction for lotus-like micro ridge
(103), (193)	Creeping/Transverse	Channel/Groove	–/–	Pressures below Capillary pressure lead to superhydrophobic state
(153)	Creeping/(Longitudinal, Transverse)	Channel/Groove	–/–	Groove edge effects reduce effective slip
(196)	Creeping/–	Channel/Bubble mattress	Deflected/Continuity	Effective slip length decreased with Capillary number
(155)	Creeping/(Longitudinal, Transverse)	Channel/Groove	Flat/–	Found good agreement with analytical results
(164)	Creeping/Transverse	Plate/Groove	Deflected/Continuity	More accurate approximation of velocity at meniscus
(198)	Inertial/(Longitudinal, Transverse)	Channel/(Groove, Post, Hole)	Flat/No-shear	Effective slip length decreases with *Re* (except for longitudinal)
(200)	Inertial/Transverse	(Pipe, Channel)/(Groove, Rib)	Deflected/No-shear	Effective slip length decreases with *Re* (more severe for pipe)
(201)	Inertial/Transverse	Channel/Dovetail cavity	Deflected/Continuity	Lower *Re*, smaller viscosity ratio lead to better lubricant retention
(202)	Inertial/–	Channel/(Hole, Post)	Deflected/Continuity	Effective slip length decreases with *Re* (more severe for holes)

a Macro/Micro geometry: Geometry of the bulk flow and that of the superhydrophobic surface. B.C.: boundary condition at the liquid/air interface. Continuity: refers to the continuity condition of the velocity and stress on the interface.

## Non-Newtonian Fluids

The flow of non-Newtonian fluids over microstructured surfaces, i.e., either superhydrophobic or liquid-infused surfaces, has been rarely studied. There could be couple of reasons for this knowledge gap. First, the earlier applications were probably more focused on Newtonian flows, e.g., drag reduction for submerged vehicles.^203^ Second, despite almost two decades of work, the problem for the Newtonian flows is not fully addressed and there are still open areas for modeling the interface deflection effects; a possible existence of other complexities, such as surfactants,^204^ makes the problem even more challenging. Finally, the flow of non-Newtonian flows are associated with nonlinear phenomena originated form their complex nonlinear rheology,^29,38,40^ which could potentially cause additional challenges when their interaction with microstructured surfaces are considered. In other words, the strain-rate dependent viscosity of both shear-thinning and yield stress materials would be nonlinearly influenced by the stick–slip condition on a superhydrophobic surface, making the non-Newtonian flow problem even more complex. In this regard, considering a Carreau shear-thinning fluid, based on eq 2, a decrease in the shear-rate causes an increase in the flow general viscosity. On the other hand, for a Bingham viscoplastic fluid, Equation 4 shows a growth of the general viscosity when the shear-rate decreases or the yield stress increases. Therefore, the general viscosity for the yield stress and shear-thinning fluids is expected to grow on the liquid/air interface of a superhydrophobic surface, where the shear-rate decreases. On the other hand, near the groove edges and on the liquid/solid contacts of the superhydrophobic surface, due to large shear-rates, the general viscosity should decrease for these fluids.

In this section, a number of existing studies dealing with non-Newtonian flows over superhydrophobic surfaces are discussed. These studies mostly address the shear-thinning fluids interactions with superhydrophobic surfaces,^34−36,205,206^ while more recently a few studies concern the problem of viscoplastic (yield stress) materials over these surfaces.^23,32,33^

### Mathematical Modelings

#### Shear-Thinning Flows

**Figure 18:**
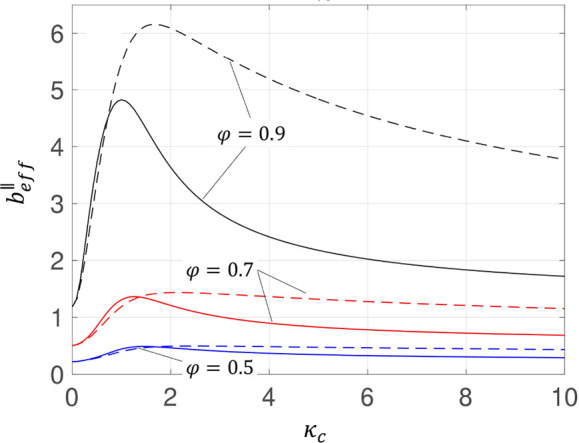
Slip length versus the Carreau number (κ_*c*_) for different widths of the liquid/air interface. Here, the interface is flat and *l* = 1. Solid and dashed lines represent the results for whole blood and Xanthan gum, respectively. Adapted from Ray et al.^34^ with permission under a Creative Commons CC BY 4.0 DEED. Copyright 2023 Elsevier.

A first-order correction to the effective slip length of a weakly shear-thinning Carreau–Yasuda fluid over superhydrophobic surfaces was performed by Crowdy.^36^ The author considered semi-infinite shear flows in longitudinal and transverse configurations, while assuming flat liquid/air interfaces with no-shear condition. Using generalized forms of the standard reciprocal theorem for Stokes flow, the solution was developed through introducing explicit integrals. It was found that, for both longitudinal and transverse flows and fixed values of the no-shear fraction and power-law index, the shear flow experiences the maximum effective slip at a critical shear rate. In addition, the author reported larger effective slip lengths for the longitudinal flow compared to the transverse one, for a given shear-thinning fluid. More recently, Ray et al.^34^ derived integral expressions to study weakly shear-thinning flows over superhydrophobic surfaces in a longitudinal configuration. The Carreau constitutive equations was used to model the shear-thinning rheology while the superhydrophobic surface was simulated by arrays of deflected liquid/air interface with no-shear condition and liquid/solid no-slip contacts. To model the strongly shear-thinning effects, the authors implemented numerical computations, where the Carreau model parameters were chosen based on the existing measurements of the whole blood and Xanthan gum solutions. They concluded that, at low Carreau numbers, the required large shear to increase the shear-thinning effects (and thus to enhance the flow-rate) can provided by the superhydrophobic surfaces, since, near the groove edges, large shear gradients were generated due to the sudden transition between the no-shear and no-slip boundary conditions. On the other hand, at large Carreau numbers, the shear rate generated in conventional no-slip channels was sufficiently large to promote the shear-thinning effects and the flow-rate; thus, the superhydrophobic surfaces were less advantageous. Due to these effects, as shown in Figure 18, at an intermediate Carreau number, a peak for the slip length was observable.

#### Viscoplastic Flows

**Figure 19:**
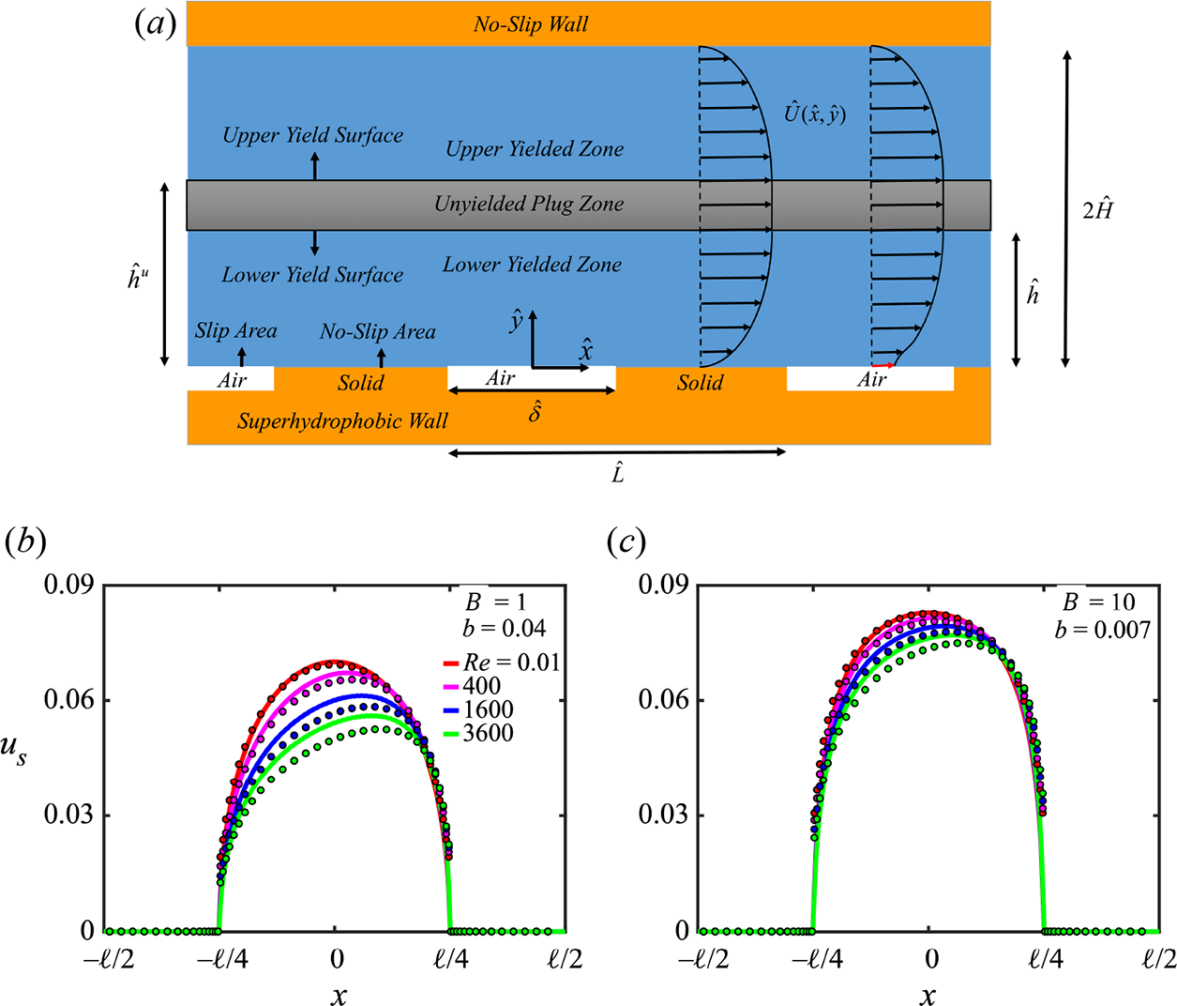
(a) Schematic of the transverse flow and (b,c) slip velocity profiles at different Bingham and Reynolds numbers. In panels b and c, *l* = 0.2, and φ = 0.5. Curves and markers represent the mathematical and numerical results, respectively. Adapted from Rahmani and Taghavi^23^ with permission under a Creative Commons CC BY 4.0 DEED. Copyright 2022 Cambridge University Press.

Recently, Rahmani and Taghavi^23^ addressed the Poiseuille flows of complex viscoplastic fluids in superhydrophobic channels with a groovy wall in transverse configuration; the flow schematic of their consideration is shown in Figure 19. The authors developed a perturbation analysis solution for the nonlinear momentum balance equations, while employing the Fourier series expansions assuming the flow periodicity transverse to the groove direction. Using arrays of no-slip and slip conditions on the superhydrophobic wall, i.e., representing the liquid/solid and a flat liquid/air interface, respectively, the Fourier coefficients were calculated through an analytical method. To model the slip condition on the liquid/air interface, the Navier slip law with a constant local slip number was used. For the assumed Bingham viscoplastic flow, the product of the defined slip number and the general (apparent) viscosity generated the slip length. Assuming a thick channel limit (i.e., ), the creeping and inertial flows were addressed to the first order of perturbations. Assuming a fixed flow rate, the authors demonstrated a growth in the effective slip length with an increase in the slip number and the Bingham number (i.e., the yield stress value). In addition, it was shown that an increase in the Reynolds number induced an asymmetry in the velocity profiles, while decreasing the dimensionless slip velocity and the effective slip length (see Figure 19). At a critical value of the slip number (i.e., *b*_*cr*_), when the no-shear condition was locally met on the liquid/air interface, the formation of an unyielded plug zone on the flat meniscus was reported. An explicit relation was developed to predict such critical slip numbers (shown in eq 19) for the creeping flow (showing a good match with the corresponding exact values):
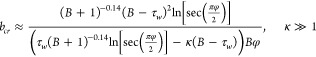
19where *B* is the Bingham number,  is the wavenumber of the superhydrophobic wall,  is the liquid/air interface fraction, and τ_*w*_ represents the wall shear stress for the classic no-slip Poiseuille-Bingham flow. The authors also compared results of their developed model with those of finite volume numerical simulations and found good agreements.

### Numerical Simulations

#### Shear-Thinning Flows

**Figure 20:**
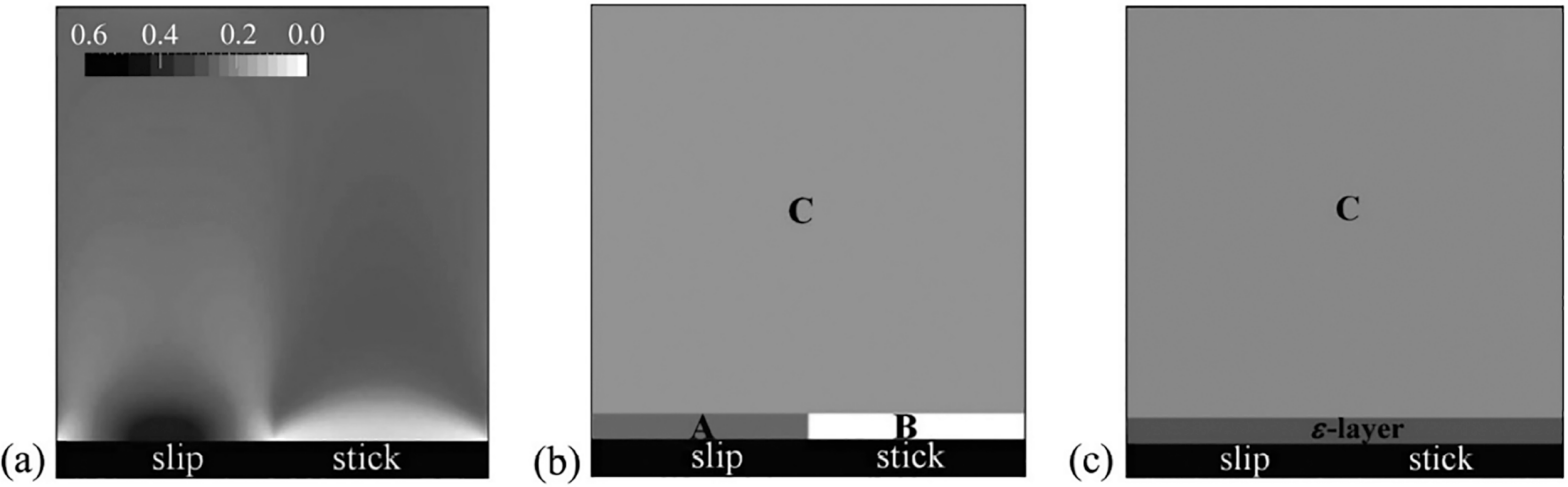
Relative viscosity field: (a) numerical simulation results, (b) average viscosity in three domains A, B and C, and (c) averaged two layer structure. Adapted from Patlazhan and Vagner^205^ with permission. Copyright 2017 American Physical Society.

Patlazhan and Vagner^205^ numerically studied the apparent slip of shear-thinning fluids in microchannels with superhydrophobic groovy walls in a transverse configuration. The authors studied an inelastic shear-thinning fluid for which the Carreau–Yasuda model described the fluid viscosity. They used the OpenFOAM package with the finite volume approach to solve the momentum balance equations. Inspired by the results of numerical computations, they separated the flow to three regions including the core region (base flow characteristics), and the stick and slip thin regions. The combination of stick and slip thin regions creates a thin ε-layer with the thickness of *d̂* (Figure 20).

Simplifying the flow domain to three regions allowed introducing a separate viscosity for each region and, with the use of some simple averaging, the viscosity for the ε-layer was obtained:^205^
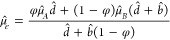
20where φ is the fraction of liquid/gas interface,  and  are the viscosity of regions A and B respectively, and *b̂* represents the local slip length at liquid/gas interface. The effective slip length for the ε-layer was obtained as
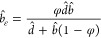
21For a given fluid and superhydrophobic surface, the authors reported a maximum apparent slip length at an intermediate value of the shear rate. Such a maximum apparent slip length was larger for the fluid with a stronger shear-thinning effect, i.e., smaller *n*.

**Figure 21:**
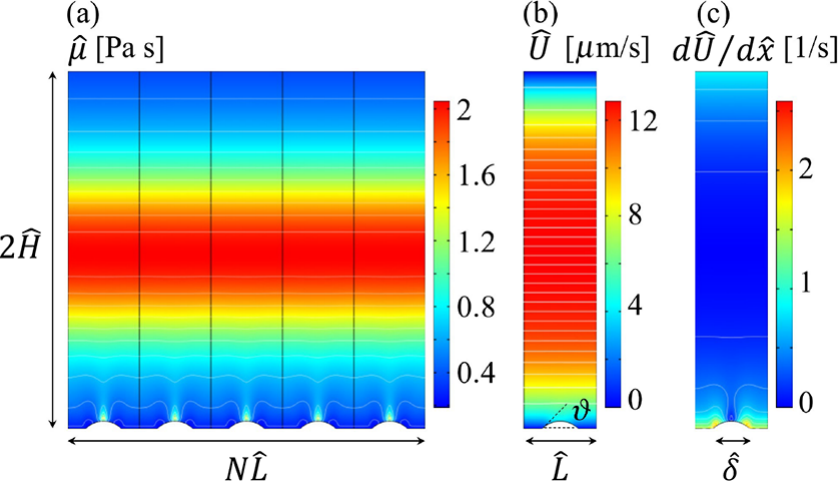
Schematic of geometry and simulation result of Haase et al.:^35^ contours of apparent viscosity (a), velocity magnitude (b), and streamwise velocity gradient (c). In this figure, *L̂* represents the period of bubble mattress structure, δ̂ is the length of each bubble, and ϑ is the bubble protrusion angle representing the meniscus angle. Adapted from Haase et al.^35^ with permission under a Creative Commons CC BY 4.0 DEED. Copyright 2017 American Physical Society.

Haase et al.^35^ performed numerical simulations of a pressure-driven flow of a shear-thinning fluid (Xanthan gum) over a bubble mattress, for transverse flow configuration. The bubble mattress configuration represented a superhydrophobic surface on which the no-slip walls and no-shear gas bubbles were transversely positioned. The authors considered a periodic two-dimensional channel with a superhydrophobic bubble mattress structure for the wall and performed numerical simulations of the flow using the COMSOL Multiphysics software. They found a general increase in the effective slip length for a shear-thinning fluid in comparison with a Newtonian fluid. For a 0.2 wt % Xanthan solution and the power-law index of *n* = 0.4, the calculated wall slip was reported to be 3.2 times larger than that of a Newtonian fluid. In Figure 21, the schematic of their geometry along with an example of the simulation results are presented.

As shown in Figure 21, the apparent viscosity grew at the top of the bubble, where the strain rate magnitude was minimum. The authors reported a maximum value for the effective slip length at an intermediate value of the flow average velocity, which was larger for the fluid with stronger shear-thinning behavior (i.e., larger wt % of Xanthan solution). In other words, the smaller power-law index (*n*) caused a larger wall-slip enhancement factor.^35^

Gaddam et al.^206^ numerically studied the flow of shear-thinning fluids in channels with superhydrophobic walls, implementing a finite volume approach. The friction factor on superhydrophobic surfaces with different topological features, i.e., textured with posts, holes, longitudinal and transverse grooves, was calculated. Similar to the case of Newtonian fluids, the obtained friction factors for the longitudinal grooves and posts were significantly smaller than those for the transverse grooves and holes. For all the studied configurations, a nonmonotonic behavior was reported for the friction factor versus the Carreau number. It was also found that the friction factor was minimum at a constant Carreau number, irrespective of the microchannel constriction ratio (i.e., the half-channel height over the periodic cell size); however, such a minimum was shifted to a larger Carreau number when the power-law index and the fraction of liquid/air interface increased.

#### Viscoplastic Flows

**Figure 22:**
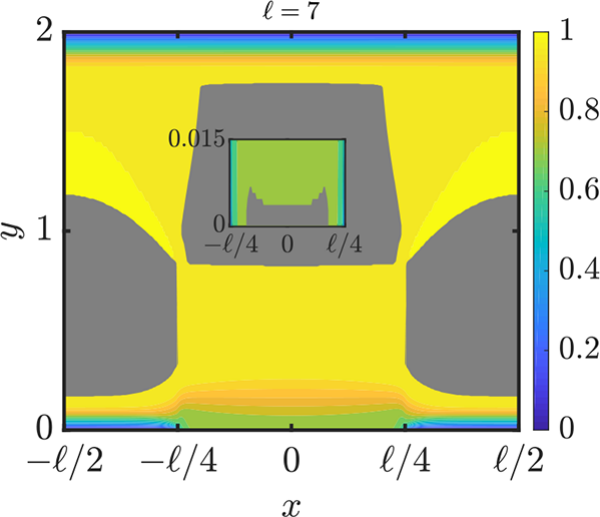
Example of the flow simulation for a thin channel showing both SH wall plug formation and the center plug breakage. Adapted from Rahmani and Taghavi^32^ with permission. Copyright 2023 Elsevier.

More recently, Rahmani and Taghavi^32^ developed finite volume numerical simulations to address the Poiseuille flow of Bingham fluids in channels with a lower superhydrophobic groovy wall. Assuming a transverse flow configuration, the authors addressed the complex interactions between the nonlinear yield stress rheology and the wall superhydrophobicity while focusing on thin channels (where the groove period was much larger than the half-channel height). Flat liquid/air interfaces along with liquid/solid contacts were assumed to simulate the superhydrophobic surface, where the slip on the liquid/air interface was modeled through the Navier slip law with a constant local slip number. The Papanastasiou regularization model was used to simulate the viscoplastic rheology of the Bingham fluid. A growth in the slip velocity was observed when the slip and Bingham numbers increased. For a sufficiently large slip number, where the no-shear condition was first met on the liquid/air interface, the formation of an unyielded plug zone (called the SH wall plug) was reported, i.e. confirming the predictions of their earlier model presented in Rahmani and Taghavi.^23^ Therefore, with an increase in the slip number, the effective slip length was first increased and eventually showed a converging trend once the SH wall plug appeared. Before any SH wall plug formation, for a given slip number and fixed other parameters, an increase in Bingham number led to a growth of the effective slip length; however, when the SH wall plug was present, an inverse trend was observed. The authors also demonstrated that the center unyielded plug zone (which is a characteristic of the yield stress channels flows) would deform and eventually break when the slip number became sufficiently large. Such a breakage was only observed for sufficiently thin channels; for thick channels the center plug would not break and its lower yield surface was shown to remain straight. An example of their flow simulation for a thin channel, where the groove period is 7 times larger than the half-channel height (*l* = 7), and the SH wall plug appears while the center plug is broken, is shown in Figure 22. In this figure, the contours of the streamwise velocity (i.e., in the *x* direction) normalized by the maximum velocity is shown (in colors), along with the unyielded plug zones (shown in gray). The SH wall plug is shown by an inset that magnifies an area on the liquid/air interface, which is located at −*l*/4 < *x* < *l*/4.

In another study, Rahmani et al.^33^ evaluated the inertial effects on the dynamics of a Poiseuille-Bingham flow in superhydrophobic thin channels. A transverse flow configuration was assumed and the channel was equipped with a lower superhydrophobic groovy wall, on which flat liquid/air interfaces were considered. The developed numerical code by Rahmani and Taghavi^32^ was utilized to address the role played by inertial through taking into account the Reynolds numbers up to *R* = 1000. Similar to the thick channel regime,^23^ an increase in the Reynolds number decreased the effective slip length and induced an asymmetry to the velocity profiles. It was also found that an increase in Reynolds number had a nonmonotonic effect on the center plug yielding and breakage, which was associated with a competition between two simultaneous inertial effects, i.e., hindering the superhydrophobic wall induced perturbations and imposing an asymmetry to the velocity profiles. In addition, an increase in the Reynolds number led to the formation of the SH wall plug at a smaller slip number, while the friction factor was enhanced.

A brief summary of the mathematical and numerical efforts conducted for the shear flows of non-Newtonian fluids over superhydrophobic surfaces is presented through Table 3.

**Table 3:** Summary of the Modeling Efforts Conducted for Non-Newtonian Flows^a^

Ref. no./method	Flow regime/type	Macro/Micro geometry	Interface/B.C.	Key finding
(36)/Math	Creeping/(Longitudinal, Transverse)	Plat/Slot	Flat/No-shear	Maximum effective slip at a critical shear-rate
(34)/(Math, Num)	Creeping/Longitudinal	Plate/Groove	Deflected/No-shear	Maximum effective slip at an intermediate Carreau number
(23)/(Math, Num)	(Creeping, Inertial)/Transverse	Channel/Groove	Flat/Local slip number	Plug zone formation on the meniscus
(205)/Num	Creeping/Transverse	Channel/Groove	Flat/No-shear	A model for Effective slip length was obtained
(35)/Num	Creeping/Transverse	Channel/Bubble mattress	Deflected/No-shear	Larger effective slip for stronger shear-thinning behavior
(32)/Num	Creeping/Transverse	Channel/Groove	Flat/Local slip number	Center plug breaks at large slip numbers
(33)/Num	(Creeping, Inertial)/Transverse	Channel/Groove	Flat/Local slip number	Formation of center plug islands due to Reynolds effects

a Macro/Micro geometry: Geometry of the bulk flow and that of the superhydrophobic surface. B.C.: boundary condition at the liquid/air interface. Math: Mathematical. Num: Numerical.

## Summary, Critical Remarks, and Future Works

### Summary

A review on the mathematical and numerical models developed for the shear flows of Newtonian and non-Newtonian fluids over superhydrophobic surfaces was conducted. The purely shear flows over a superhydrophobic surface and pressure-driven flows in channels with one or two superhydrophobic walls fabricated by adding micro scale groove, posts and holes were the main configuration studied. In general, with respect to the groovy superhydrophobic surfaces, the pressure gradient and groove directions were considered to make different angles, θ = 0, 0 < θ < 90°, and θ = 90°, forming the longitudinal, oblique, and transverse flows, respectively. Considering the air pockets trapped inside the cavities formed on the superhydrophobic surfaces, the working liquid interface with the trapped air showed different states, playing a large role on the overlying flow dynamics.

The early works developed a mathematical framework to address the Newtonian flows over surfaces with mixed no-shear and no-slip boundary conditions. Such works considered a state of the superhydrophobic surface where a flat liquid/air interface was assumed, while showing no-shear condition, and was later used by several researchers to develop extended models for more complex states of superhydrophobic and liquid-infused surfaces.

A series of mathematical models based on assuming a constant local slip length for flat liquid/air interfaces were later developed to account for the oblique flow configuration of Newtonian flows by defining a rotation tensor. Subsequently, the constant local slip length model theory were later revisited through developing a tensorial slip length, i.e., considering different local slip lengths for the longitudinal and transverse flows, while introducing a smooth distribution for the local slip length in the transverse direction, i.e., normal to the groove direction. Considering a constant shear stress condition on the liquid/air interface, a new tensorial slip length model with smooth spatial distributions for the local slip length was also introduced. Taking into account the meniscus curvature effects on the overlying flow dynamics in the developed models was indeed a breakthrough. This new modeling approach consists of perturbation analysis techniques, domain mappings, and connecting the inner cavity and outer liquid flows, allowed addressing the curvature effects of the deflected liquid/air interface on the overlying flow dynamics through evaluating the alterations made on the effective slip length, frictions factor and the flow rate. There have been also attempts to address inertial flows of Newtonian fluids over superhydrophobic surfaces, i.e., through conducting perturbation and linear stability analyses, leading to finding new modes of instability due to the presence of superhydrophobic surfaces.

Regarding mathematical models developed for non-Newtonian flows over superhydrophobic surfaces, very few studies were developed. Perturbation analyses led to obtaining integral expressions for the flow of shear-thinning fluids for both flat and deflected meniscus. To address the yield stress effects on the creeping and inertial viscoplastic flows in superhydrophobic channels, perturbation analyses along with Fourier expansion methods were utilized. The aforementioned attempts relied on either no-shear or constant local slip condition at the liquid/air interface.

Numerical simulations for Newtonian flows over superhydrophobic surfaces were conducted using finite volume/element, molecular dynamics, dissipative particle dynamics, and lattice Boltzmann methods. Considering the continuum models, the first numerical attempts were initiated through considering only the liquid phase, while modeling the liquid/air interface (flat or deflected) as no-shear or partial-shear boundary conditions. Considering both the liquid and gas (cavity fluid) phases, different numerical methods were developed to account for the cavity flow, while modeling its effects on the meniscus deflection and the overlying flow dynamics. This includes domain decomposition methods and nanoscale phase field simulations. Molecular dynamics simulations were also conducted to address the intricate interfacial dynamics near the liquid/air interface. On the other hand, the mesoscale simulations through developing models based on dissipative particle dynamics and lattice Boltzmann methods were used more frequently; this can be associated with their less computational costs compared to the molecular dynamics simulations. Using numerical simulations, inertial flows of Newtonian fluids over superhydrophobic surfaces have been studied during the past few years. In some of the developed models, the liquid/air interface was treated as a flat slippery meniscus. On the other hand, in some other studies, the liquid/air interface deflection was taken into account. Numerical simulations of non-Newtonian flows over superhydrophobic surfaces were conducted in a couple of works, by employing the finite volume method, to develop single-phase modelings with no-shear and partial-shear conditions at the flat and arc-shaped deflected menisci.

### Critical Remarks

Based on the provided literature review, there are critical aspects about the modeling of Newtonian and non-Newtonian shear flows over superhydrophobic surfaces. We discuss such critical aspects separately for Newtonian and non-Newtonian flows:

#### Newtonian Flows

- In spite of several significant contributions on quantifying the local slip length (i.e., the slip condition on the liquid/air interface), this problem seems to be still an open challenge. Such a local slip length can be affected by many parameters, e.g. the microstructure geometry, the dynamics of the liquid/air interface, the inner cavity flow and the bulk flow dynamics, the flow three-dimensionality, and so on.
- The singularity at the transition point (line) between the slip and no-slip regions is an important consideration. Although a few methods have been developed to treat such a singularity, it is still not clear how such a treatment can affect some aspects of the flow dynamics, e.g. the flow stability picture.
- Considering superhydrophobic channels, two-dimensional plane channel flows were mainly considered, although, in many practical applications, the effects of channel side walls are remarkable and the flow is three-dimensional. In the other words, the cross-sectional aspect ratio of the channel is usually not large enough to allow assuming a two-dimensional plane channel flow.
- Regarding the deflected liquid/air interface, in many recent works, a circular arc shape was considered for the interface. In fact, this is an idealistic assumption that requires a uniform distribution of the pressure and surface tension on the liquid/air interface.
- In several recent works, the flow stability on a superhydrophobic wall were studied while assuming a flat liquid/air interface. In such studies, an important point is that the liquid/air interface deflection might remarkably alter the flow stability picture. Therefore, the complex interaction between the flow perturbation field and the liquid/air interface dynamics should be a major consideration. The same point applies to the surfactant-contaminated flows over superhydrophobic surfaces, where the interaction between the surfactant load and distribution and the deflected meniscus is of high importance.

#### Non-Newtonian Flows

The critical aspects discussed for the Newtonian flows also apply to the non-Newtonian counterpart. However, there are unique aspects regarding the non-Newtonian flow dynamics over superhydrophobic surfaces which we can mention for the shear-thinning and viscoplastic rheology:

- The theoretical modeling of shear-thinning flows over superhydrophobic surfaces are limited to weakly shear-thinning behavior. The nonlinear complexity added by the strong shear-thinning rheology is an important consideration for extending such models.
- Considering both shear-thinning and viscoplastic materials, the fluid rheology is a key factor when quantifying the local slip length on the liquid/air interface, i.e. in addition to the above-mentioned parameters for the Newtonian flows. In this regard, the spatially variable viscosity on the interface would affect the distribution of the local slip length.
- Regarding the viscoplastic flow dynamics over superhydrophobic surfaces, the developed models are limited to the onset of the plug formation at the liquid/air interface. The big challenge is that, after such a plug formation, the perturbation theory would not be valid, i.e. the plug zone cannot be perturbed since it must show a solid-like behavior with zero deformation. Another important challenge is that the boundary of the formed plug zone is not known *a priori*, such that one could limit the perturbation field within the fully yielded zone.
- Considering the viscoplastic problem in thin channels, the center plug is highly affected by the superhydrophobic wall, leading to its severe deformation. Considering a highly deformed center plug, developing theoretical models would be a challenging task.
- Numerical simulations of the viscoplastic flows over superhydrophobic surfaces were conducted using a regularization method. However, it is well-known that the viscoplastic flow simulation using the augmented Lagrangian methods (ALMs) is capable of providing more accurate predictions of the yield surfaces. Considering the complexities added by the superhydrophobic walls and the slow nature of simulations using ALMs, the viscoplastic flow simulations over superhydrophobic surfaces based on ALM algorithms would be computationally expensive and challenging.

### Future Works

Some of current trends and future works regarding shear flows of Newtonian over superhydrophobic surfaces can be highlighted as

- Developing sophisticated two-phase flow models with considering a deformable meniscus for both laminar and turbulent flows.
- Considering new complexities in the problem by adding different agents to the fluid, e.g., surfactants.
- Heat transfer enhancement for flows over superhydrophobic surfaces.

On the other hand, there is a serious knowledge gap regarding our understanding of non-Newtonian shear flow over superhydrophobic surfaces. A few potential future directions could be highlighted as

- Developing models to address the intricate interaction between the superhydrophobicity and the nonlinear fluid rheology, e.g., shear-thinning and thickening, viscoplastic, viscoelastic, thixotropy, etc.
- Addressing the complex liquid/air interface dynamics concerning the meniscus deflection and interaction with the non-Newtonian rheology.
- Addressing turbulent flows of non-Newtonian fluids over superhydrophobic surfaces.
